# Transferrin restrains rather than drives neuronal ferroptosis after intracerebral haemorrhage

**DOI:** 10.3389/fphar.2026.1945504

**Published:** 2026-10-28

**Authors:** Zhen Liu, Xuehan Zhuang, Bo Feng, Geyan Tian

**Affiliations:** 1 Department of Neurology, The Affiliated Hospital of Yangzhou University, Yangzhou University, Yangzhou, Jiangsu, China; 2 School of Traditional Chinese Medicine, Faculty of Medicine, Yangzhou University, Yangzhou, Jiangsu, China; 3 Laboratory of Intensive Care, The Affiliated Hospital of Yangzhou University, Yangzhou University, Yangzhou, Jiangsu, China

**Keywords:** apo-transferrin, ferroptosis, intracerebral haemorrhage, iron buffering, transferrin

## Abstract

**Introduction:**

The transferrin‐transferrin receptor axis is the principal route by which neurons acquire iron, and it is generally regarded as a driver of ferroptosis: transferrin receptor 1 is used as a ferroptosis marker, and blocking transferrin‐bound iron uptake is protective in several models of iron‐dependent injury. That framework predicts that transferrin should be harmful after intracerebral haemorrhage (ICH), a condition defined by catastrophic iron overload. Clinical observations point the other way, since patients with higher circulating transferrin have smaller haematomas and better outcomes

**Methods:**

We resolved this contradiction using bidirectional, neuron‐targeted manipulation of transferrin in a rat autologous‐blood model of ICH. Transferrin was silenced or overexpressed in neurons with adeno‐associated virus given 3 weeks beforehand, the same manipulations were applied to hemin-exposed differentiated PC‐12 neurons, transcriptomes were sequenced, and recombinant apo‐transferrin was given intraperitoneally 1 h after ICH at 4, 10 or 20 mg/kg.

**Results:**

Endogenous transferrin increased two‐ to threefold in perihaematomal neurons and microglia, peaking on day 3, the same time point at which ferroptotic injury was maximal. Silencing neuronal transferrin did not protect; it increased perihaematomal iron, hydrogen peroxide, and malondialdehyde; shrank neuronal mitochondria further; and enlarged the lesion. Overexpression produced the mirror image, driving iron below sham levels and halving neurodegeneration. Inversion was reproduced in hemin‐exposed PC‐12 neurons. Transcriptionally, transferrin did not reinforce antioxidant defence. It abolished the need for it. Hemin moved 6,976 genes in wild‐type neurons but only 343 in transferrin‐overexpressing ones, a 95% collapse of the injury programme. Across the 6,974 hemin‐responsive genes, the effect of transferrin ran opposite to the effect of hemin (Pearson r = −0.869), reversing 99% of the genes that both perturbations moved significantly. The hemin‐induced oxidative stress programme, including the HO‐1/SLC7A11/TXNRD1 module, returned towards baseline. Recombinant apo‐transferrin given 1 h after ICH reproduced these effects with a sharp threshold at 20 mg/kg, halved lesion volume, and restored sensorimotor and cognitive function at 14 days.

**Discussion:**

We propose that ICH inverts transferrin's function: confronted with heme‐derived iron that bypasses the transferrin cycle, transferrin is repurposed from an iron donor towards a high‐affinity extracellular iron sink. Its buffering capacity, rather than iron load itself, sets the threshold for neuronal ferroptosis. That capacity is exhaustible, saturating well below what the injured brain requires.

## 1 Introduction

Intracerebral haemorrhage accounts for 10%–30% of all strokes and kills or disables most of the million-plus people it strikes each year (van Asch et al., 2010; Wu et al., 2019). No treatment has yet been shown to alter this burden of death and disability (van Asch et al., 2010; Wu et al., 2019). The initial mechanical disruption caused by the haematoma is essentially complete within minutes, but its physical and biochemical consequences are not: red-cell lysis begins within hours, and the release of haemoglobin, heme, and iron into the surrounding parenchyma continues for days to weeks, driving oxidative damage to the tissue that survived the bleed (Keep et al., 2012; Balami and Buchan, 2012). This window is the reason iron has been the most persistently pursued target in the field.

Much of that damage is now attributed to ferroptosis, an iron-dependent death programme in which ferrous iron and hydrogen peroxide generate hydroxyl radicals through Fenton chemistry, peroxidising membrane lipids until the cell fails (Stockwell et al., 2017; Tang et al., 2021). Ferroptosis occurs early after experimental ICH, and inhibiting it protects the brain (Li et al., 2017; Wang et al., 2023). The therapeutic corollary—removal of the iron—has been tested. Deferoxamine reached a randomised phase 2 trial in ICH patients, and although it was safe and hinted at benefit in a subgroup, it did not meet its primary endpoint (Selim et al., 2019). Chelators are indiscriminate: they strip iron from the enzymes that need it as readily as from the pool that does the harm; they enter the brain poorly, and their dosing is constrained by toxicity. The difficulty may lie less in the execution than in the premise. Stripping iron out of an organ is not the only way to stop iron from doing chemistry; holding it still would do as well, and the brain already possesses a protein built for exactly that.

Transferrin (TRF) is a bilobed glycoprotein that binds ferric iron with a dissociation constant near 10^−22^ M at physiological pH and releases it only on acidification below pH 6.5 (Leitner and Connor, 2012). This is the reason essentially no free iron circulates in plasma. Yet in the ferroptosis literature, transferrin is cast not as a protector but as a supplier. In health, this framing is defensible: hepatic transferrin synthesis rises when iron stores are low and falls when they are replete, so that circulating transferrin output tracks the body’s need to deliver iron rather than any tendency to withhold it (Morton and Tavill, 1977). Transferrin-bound iron delivered through transferrin receptor 1 (TfR1) feeds the labile iron pool that ferroptosis requires; TfR1 is used as a specific marker of ferroptotic cells (Feng et al., 2020); and knocking down TfR1 lowers labile iron, lowers reactive oxygen species (ROS), and prevents ferroptosis across a range of tissues (Feng et al., 2020; Xiong et al., 2014). On that reading, transferrin should be the last thing one would want in a brain already drowning in iron, and its induction after haemorrhage, reported consistently in rodents and in patients (Wu et al., 2003; Chen et al., 2015), should mark a maladaptive response worth blocking. This is consistent with a human perihaematomal microarray study (Rosell et al., 2011), and more recently, a human *ex vivo* study showing that free iron released after intracerebral haemorrhage drives a ferroptotic transcriptional signature in perihaematomal tissue (Balk et al., 2025).

The clinical data do not cooperate. In ICH patients, serum transferrin is inversely related to haematoma volume and is substantially higher in those who recover well; together with iron and ferritin, it carries prognostic information (Yang et al., 2016). Transferrin is also upregulated by thrombin preconditioning, which reduces the brain oedema caused by lysed erythrocytes (Xi et al., 2003). Something is wrong with the simple picture: a protein said to fuel ferroptosis rises after haemorrhage, and the patients with the highest admission serum transferrin concentration do best (Yang et al., 2016).

We suspected that the contradiction is contextual rather than real. The canonical scheme was built in cells whose iron arrives, by design, as diferric transferrin destined for a receptor. A haemorrhagic brain violates each of these assumptions: iron does not arrive as diferric transferrin, it does not enter through a receptor, and its quantity is not regulated. Iron arrives as heme and non-transferrin-bound species, outside the transferrin cycle altogether, in quantities that no receptor regulates. Under those conditions, the rate-limiting property of transferrin is no longer how much iron it can deliver but how much it can hold. A protein that is only ∼30% iron-saturated under physiological conditions (Gaasch et al., 2007) is, in a haemorrhagic field, less a delivery vehicle than an unfilled sink. If that is right, transferrin’s sign should flip after ICH: the same protein that promotes ferroptosis in an iron-replete neuron should restrain it in a haemorrhagic one.

Testing this requires manipulating transferrin in both directions in the intact brain, which has not been done in ICH; the exogenous protein has been given to mice, where it improved neurobehavioural scores without reducing haemorrhage volume or moving classical ferroptosis markers (García-Serran et al., 2023), leaving both the mechanism and the role of the endogenous protein unresolved. Here, we knocked down and overexpressed transferrin in rat neurons using AAV under a neuron-specific promoter, reproduced the manipulations in hemin-exposed neurons, assessed the consequences transcriptome-wide, and asked whether recombinant apo-transferrin can be given as a drug after the bleed.

## 2 Materials and methods

### 2.1 Animals

A total of 325 male Sprague–Dawley rats (250–280 g; Huachuang Sino, Taizhou, China) were housed under a 12-h light/dark cycle at controlled temperature and humidity for at least 3 days before any procedure, with food and water available *ad libitum*. All procedures were approved by the Animal Care and Use Committee of the Affiliated Hospital of Yangzhou University (No. 202309022) and conducted in accordance with the ARRIVE guidelines. Fourteen rats were excluded from analysis because ICH induction failed by the criteria in Section 2.5.

### 2.2 Cell culture

PC-12 cells (BNCC100235; BeNa Biotechnology, China) were maintained in RPMI-1640 (G4531, Servicebio, China) with 10% fetal bovine serum (FBSST-01033, OriCell, China) and 1% penicillin–streptomycin (15140122, Gibco, United States) at 37 °C in 5% CO_2_. Highly differentiated PC-12 cells adopt a neuronal phenotype and are used here as neurons. PC-12 cells retain the core transferrin-receptor-mediated iron-uptake pathway used by neurons, which motivates their use here, but as a chromaffin-lineage line, their iron-handling kinetics may not be quantitatively identical to primary cortical neurons; this is addressed as a limitation in Discussion.

### 2.3 Experimental design

The five experiments are summarised in Figure 1. Investigators who performed procedures, collected data, and scored outcomes were blinded to allocation throughout.

**FIGURE 1:**
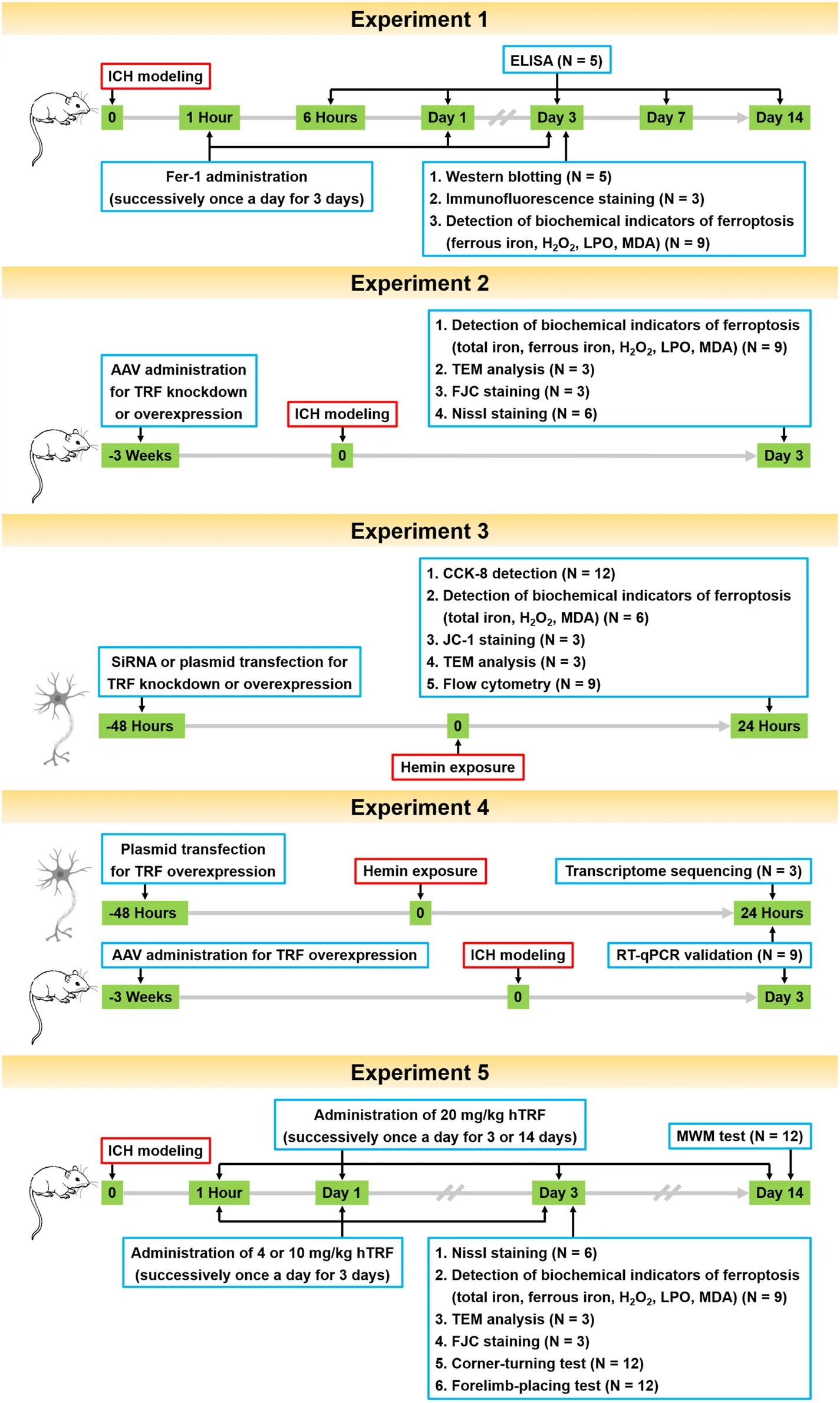
Experimental design. AAV, adeno-associated virus; CCK-8, cell counting kit-8; ELISA, enzyme-linked immunosorbent assay; Fer-1, ferrostatin-1; FJC, Fluoro-Jade C; hTRF, humanised recombinant apo-transferrin; ICH, intracerebral haemorrhage; LPO, lipid peroxide; MDA, malondialdehyde; MWM, Morris water maze; RT-qPCR, real-time quantitative polymerase chain reaction; siRNA, small interfering RNA; TEM, transmission electron microscopy; TRF, transferrin.

#### 2.3.1 Experiment 1

To characterise endogenous transferrin (TRF) after ICH, rats were randomised to a Sham group and to groups killed 6 h and 1, 3, 7, and 14 days after ICH; TRF was measured by ELISA and Western blotting. Two further groups were used for immunofluorescence to establish which cells express TRF on day 3. To confirm that ferroptosis contributes to the injury, the ferroptosis inhibitor ferrostatin-1 (Fer-1) was given after ICH and ferrous iron, hydrogen peroxide (H_2_O_2_), lipid peroxide (LPO), and malondialdehyde (MDA) were measured in the ipsilateral hemisphere on day 3.

#### 2.3.2 Experiment 2

AAV carrying either a TRF short hairpin RNA (shRNA) or the TRF gene was injected into the lateral ventricle 3 weeks before ICH. On day 3 after ICH, we measured total iron, ferrous iron, H_2_O_2_, LPO, and MDA; examined neuronal mitochondria by transmission electron microscopy (TEM); stained degenerating neurons with Fluoro-Jade C (FJC); and measured lesion volume by Nissl staining.

#### 2.3.3 Experiment 3

Small interfering RNA (siRNA) or TRF-bearing plasmids were transfected into PC-12 cells 48 h before hemin exposure. Twenty-four hours after hemin, we assayed viability, total iron, H_2_O_2_, MDA, mitochondrial membrane potential, ultrastructure, and apoptosis.

#### 2.3.4 Experiment 4

Transcriptome sequencing was performed on TRF-overexpressing PC-12 cells with and without hemin. Selected differentially expressed genes were re-measured by real-time quantitative polymerase chain reaction (RT-qPCR) in both cells and brain tissue.

#### 2.3.5 Experiment 5

Humanised recombinant apo-transferrin (hTRF; 4, 10, or 20 mg/kg) was given to rats after ICH, and lesion volume was assessed on day 3. The efficacious dose (20 mg/kg) was then carried forward for biochemistry; TEM; FJC staining; and corner-turning, forelimb-placing, and Morris water maze (MWM) tests.

### 2.4 Intracerebroventricular administration of AAV

AAV vectors driven by a neuron-specific promoter were constructed by Cyagen (Suzhou) Biosciences, China. For knockdown, 1 × 10^10^ particles of AAV-PHP.eB-hSyn > rTf [TGACTGCCCAGAAGGATTTATCTCGAGATAAATCCTTCTGGGCAGTCA]-CMV > EGFP (titre 1 × 10^13^ GC/mL) were injected stereotactically into the right lateral ventricle (1.0 mm posterior to bregma, 1.5 mm lateral, and 3.5 mm ventral) at 0.2 μL/min (Hafez et al., 2016); controls received the same quantity of AAV-PHP.eB-U6 > scramble shRNA-CMV > EGFP (2 × 10^13^ GC/mL). For overexpression, 1 × 10^10^ particles of AAV-PHP.eB-hSyn > Kozak-Rat Tf CDS [NM_001013110.1]/3 × FLAG/P2A/EGFP (5 × 10^12^ GC/mL) were injected identically with AAV-PHP.eB-CMV > EGFP (2 × 10^13^ GC/mL) used as a control. Three weeks later, a subset was killed to verify efficiency by RT-qPCR; the remainder underwent ICH.

### 2.5 Induction of ICH and drug administration

ICH was induced by the intrastriatal infusion of autologous blood (Fang et al., 2023). Rats anaesthetised with isoflurane were fixed in a stereotaxic frame, a 1-mm burr hole was drilled, and a 26-gauge cannula was used to infuse 100 μL of caudal vein blood into the right corpus striatum (0.2 mm anterior to bregma, 3.5 mm lateral, and 5.5 mm below the skull; 10 μL/min). Core temperature was held at 37 °C ± 0.5 °C during surgery and for 1 h afterwards. Only animals scoring 8–10/10 on the corner-turning test and 0–2/10 on the forelimb-placing test at 1 h entered subsequent experiments. Controls received an empty cannula under identical conditions.

Fer-1 (Cas#347174-05-4; TargetMol, United States) was dissolved in dimethyl sulfoxide (10 mg/mL), diluted in normal saline, and given intraperitoneally at 2 mg/kg 1 h after ICH and daily for 3 days (Chen et al., 2019). hTRF (Cas#11096-37-0; Solarbio, China) was dissolved in ultrapure water and given intraperitoneally at 4, 10, or 20 mg/kg (García-Serran et al., 2023; Tang et al., 2020) 1 h after ICH and daily for 3 or 14 days. Controls received an equal volume of vehicle.

### 2.6 Cell transfection

Three siRNAs were transfected separately using Lipofectamine 3000 (L3000015, Invitrogen, United States) per the manufacturer’s instructions; efficiency was confirmed by RT-qPCR at 48 h, and all three performed comparably. Sequences were as follows: siRNA-1, 5′-GGA​AAG​ACC​UGC​UGU​UUA​ATT-3′/5′-UUA​AAC​AGC​AGG​UCU​UUC​CTT-3′; siRNA-2, 5′-GGA​ACA​ACC​UGA​AAG​GCA​ATT-3′/5′-UUG​CCU​UUC​AGG​UUG​UUC​CTT-3′; siRNA-3, 5′-GAA​CAG​ACU​UCC​AGC​UGA​ATT-3′/5′-UUC​AGC​UGG​AAG​UCU​GUU​CTT-3′. TRF-bearing plasmids were transfected identically. siRNAs and plasmids were synthesised by Wuhan GeneCreate Biological Engineering, China.

### 2.7 *In vitro* ICH model

Hemin (Cas#16009-13-5, TargetMol) was dissolved in 40 g/L NaOH, diluted in distilled water to 10 mmol/L, and further diluted in RPMI-1640 to a 100 μmol/L working solution; cells were exposed for 24 h (Zille et al., 2017).

### 2.8 ELISA

TRF in brain tissue ipsilateral to the haematoma was measured with a kit from Elabscience (E-EL-R3028, China) per the instructions.

### 2.9 Western blotting

Proteins from perihaematomal tissue were separated by SDS-PAGE, transferred to PVDF, and blocked in 5% bovine serum albumin (Fang et al., 2023). Membranes were incubated overnight at 4 °C with antibodies against β-actin (1:3000; GB15003, Servicebio) or TRF (1:1000; 17435-1-AP, Proteintech, United States) and then with HRP-conjugated IgG (H + L) (1:5000; SA00001-2, Proteintech) for 1 h at room temperature. Signals were developed by enhanced chemiluminescence, quantified in ImageJ, and normalised to β-actin. Uncropped membranes for the blots shown in Figure 2B are provided in Supplementary Material 2.

**FIGURE 2:**
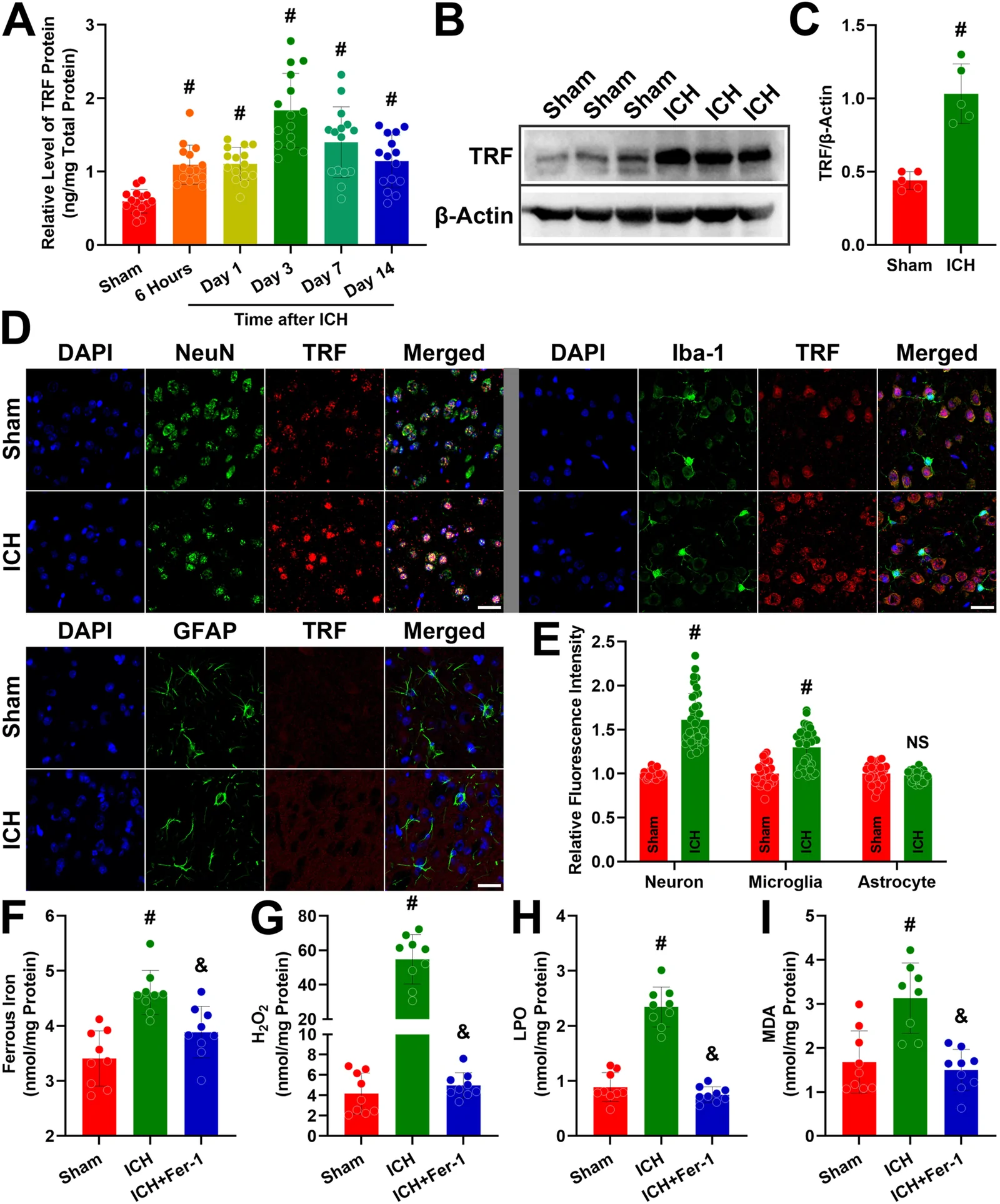
TRF increases in the brain after ICH, and ferroptosis is a major cause of neuronal death post-ICH: **(A)** Quantification of TRF. N = 5 with three replicates. **(B)** Representative bands of Western blotting. **(C)** Western blotting analysis of TRF. N = 5. **(D)** Representative images of immunofluorescence staining for TRF cellular expression. DAPI, marker of the cellular nuclei; GFAP, marker of astrocytes; Iba-1, marker of microglia; NeuN, marker of neurons. Scale bar = 30 μm. **(E)** Quantification of the fluorescence intensity of the TRF signal. N = 3. Sham, group subjected to sham operation; ICH, group subjected to ICH operation. #, *p* < 0.05 versus Sham; NS, no significance versus Sham. **(F–I)** Quantification of ferrous iron, H_2_O_2_, LPO, and MDA, respectively. N = 9. Sham, group subjected to sham operation and vehicle administration; ICH, group subjected to ICH operation and vehicle administration; ICH + Fer-1, group subjected to ICH operation and Fer-1 administration. #, *p* < 0.05 versus Sham; &, *p* < 0.05 versus ICH. All data were expressed as the mean ± SD.

### 2.10 RT-qPCR

RNA from perihaematomal tissue or PC-12 cells was extracted with TRNzol Universal Reagent (DP424, TIANGEN, China) (Zhang et al., 2025). One microgram was reverse-transcribed with PrimeScript RT Master Mix (RR036A, TaKaRa, Japan) and amplified with Hieff qPCR SYBR Green Master Mix (11201ES08, YEASEN, China) using a CFX96 system (Bio-Rad, United States). Expression was normalised to β-actin. Primers are listed in Supplementary Table S1.

### 2.11 Immunofluorescence staining

Brains were fixed, dehydrated, and cut into 8 μm coronal sections (Sun et al., 2020). Sections were incubated overnight at 4 °C with antibodies against TRF (1:50), NeuN (1:500; GB15138, Servicebio), Iba-1 (1:500; GB15105, Servicebio), or GFAP (1:500; GB12100, Servicebio) and then for 1 h at 37 °C with Alexa Fluor 488- and 594-conjugated IgG (H + L) (1:500; A-11001 and A-11012, Invitrogen) and counterstained with DAPI (C1006, Beyotime, China) for 5 min. Images were captured on a Nikon A1 HD25 confocal microscope. Three sections spanning the haematoma, at least 100 μm apart, were selected at random per rat; four non-overlapping fields per section, scanned at identical settings, were quantified in ImageJ and normalised to Sham. For NeuN, Iba-1, and GFAP, a mask was drawn around marker-positive cells in each field, and TRF fluorescence intensity within that mask was measured and expressed relative to the mean Sham value; the fold-changes reported for Figure 2E are therefore intensity ratios within these masks, not counts of double-positive cells.

### 2.12 Biochemical assays

Total iron, ferrous iron, H_2_O_2_, LPO, and MDA were measured in perihaematomal tissue (dissected after transcardial perfusion with normal saline) and in cell pellets using E-BC-K772-M (Elabscience), E-BC-K773-M (Elabscience), S0038 (Beyotime), BC5245 (Solarbio), and BC0025 (Solarbio), respectively, per the manufacturers’ instructions.

### 2.13 TEM

Basal ganglia ipsilateral to the haematoma were trimmed to ∼1 mm^3^ blocks; cells were collected as pellets (Chen et al., 2019). Samples were fixed in 2.5% glutaraldehyde for 2 h at 4 °C, post-fixed in 1% osmium tetroxide, dehydrated in ethanol, infiltrated with propylene oxide, embedded in Epon-812, cut at 60 nm, and counterstained with uranyl acetate and lead citrate. Mitochondria were imaged using a Hitachi HT7800 microscope, and their cross-sectional area was measured using ImageJ.

### 2.14 FJC staining

FJC staining was performed with the Degenerating Neurons Stain Kit (G3262, Solarbio) per the instructions. Four non-overlapping fields in each of three sections spanning the haematoma were counted using ImageJ.

### 2.15 Nissl staining

Nissl staining was performed with the Nissl Stain Kit (G1086, Servicebio). Slides were scanned using a Pannoramic MIDI scanner. Lesion areas were measured using ImageJ on two sections encompassing the core haemorrhage and separated by 1 mm; areas were averaged and multiplied by 1 mm to give lesion volume in mm^3^.

### 2.16 JC-1 staining

JC-1 staining was performed with the JC-1 Mitochondrial Membrane Potential Assay Kit (40706ES60, YEASEN). Images were captured using a Nikon A1 HD25 confocal microscope, and fluorescence was quantified using ImageJ as the aggregate/monomer ratio.

### 2.17 Cell viability

Cell viability was measured with cell counting kit-8 (C0040, Beyotime). Viability (%) = [OD_experimental − OD_blank]/[OD_normal control − OD_blank] × 100.

### 2.18 Flow cytometry

Apoptosis was assessed with the Annexin V-FITC/PI Apoptosis Detection Kit (40302ES60, YEASEN); at least 10,000 events were recorded per sample.

### 2.19 Transcriptome sequencing

RNA was extracted from wild-type and transferrin-overexpressing PC-12 cells, with and without 24 h hemin exposure (100 μmol/L, as described above), giving four groups (WT, WT + Hemin, TRF-OE, and TRF-OE + Hemin) with three independent biological replicates each. Hemin was used in preference to a ferric salt because it is the form in which iron actually reaches the neuron after a haemorrhage: haemoglobin released by erythrocyte lysis is oxidised and dissociates to heme, which is degraded intracellularly by heme oxygenase-1 to liberate ferrous iron. A ferric salt would deliver iron in a form that transferrin binds directly and would prejudge the question the experiment was designed to ask. Sequencing was performed by using Illumina HiSeq 4000 (Wuhan Servicebio Technology, China). Transcript levels were normalised as transcripts per million (TPM). Differential expression was called with DESeq2 (Love et al., 2014), and P-values were corrected by the Benjamini–Hochberg procedure; the threshold was an adjusted P < 0.05 with |log2(fold change)| ≥ 1, applied identically to all five pairwise comparisons. Differentially expressed genes were mapped to Gene Ontology (GO) terms (Ashburner et al., 2000) and to KEGG pathways in KOBAS (Xie et al., 2011).

To ask whether transferrin reverses the hemin programme rather than merely damping it, we defined the hemin-responsive gene set as all genes meeting the above threshold in the WT + Hemin versus WT comparison, and for each such gene, we compared its log2(fold change) under hemin with its log2(fold change) attributable to transferrin in the hemin-exposed state (TRF-OE + Hemin versus WT + Hemin). Association between the two was quantified by Pearson and Spearman correlation, and a gene was scored as reversed when the two values carried opposite signs. This analysis reuses the differential-expression output and introduces no additional testing of individual genes. Because the two contrasts share the hemin-exposed wild-type samples, three controls were applied: a split-sample estimate in which the hemin effect was computed from two hemin-exposed replicates and the transferrin effect from the third, so that the two estimates shared no sample (rotated over all three choices, restricted to genes with mean normalised counts ≥ 10); a label-permutation null of 2,000 iterations; and a comparison of two contrasts sharing no samples at all—the effect of hemin in wild-type cells against the effect of hemin in transferrin-overexpressing cells. The scripts that regenerate every count, correlation, control, and supplementary figure reported here are provided with the paper.

### 2.20 Neurobehavioural tests

Corner-turning and forelimb-placing tests were performed on day 3 to assess sensorimotor function and the MWM on day 14 to assess motor coordination and cognition (Liu et al., 2018). In the corner-turning test, rats entered a 30° corner and exited by turning left or right; 10 trials were run at ≥30 s intervals, and right turns were counted, with only turns involving full rearing along a wall included. In the forelimb-placing test, each forelimb was tested independently by brushing the ipsilateral vibrissae against a countertop edge, and timely placements over 10 trials were counted. In the MWM, rats were released from semirandom start positions and given 120 s to find a visible platform; in the subsequent probe trial, the platform was removed, and escape latency to the target quadrant, crossing frequency, and swim path were recorded.

### 2.21 Statistics

Power was calculated using GraphPad StatMate at α = 0.05 and 80% power. Analyses were performed by GraphPad Prism using one-way ANOVA with Tukey’s multiple comparisons test. Data are presented as the mean ± SD; *p* < 0.05 was considered significant.

## 3 Results

### 3.1 Endogenous transferrin rises in neurons and microglia as ferroptotic injury peaks

Transferrin in the ipsilateral hemisphere was already elevated 6 h after ICH, climbed to a peak on day 3 at roughly three times the Sham level, and remained above baseline through day 14 (Figure 2A). Western blotting on day 3 confirmed the increase (Figures 2B,C). Double immunofluorescence localised induction: the TRF signal intensified in NeuN-positive neurons (1.6-fold) and, less markedly, in Iba-1-positive microglia (1.3-fold), while GFAP-positive astrocytes were unchanged (Figures 2D,E). Whatever role transferrin is playing after ICH, it is doing it in the two cell types that handle heme-derived iron. That the injury it accompanies is ferroptotic was confirmed pharmacologically. On day 3, ferrous iron, H_2_O_2_, LPO, and MDA were all sharply raised in the ipsilateral hemisphere, and all four were brought back towards Sham values by Fer-1 (Figures 2F–I). The peak of the transferrin response therefore coincides with the peak of iron-dependent lipid peroxidation. Two readings of that coincidence are possible, and they are opposites: transferrin is delivering the iron that kills the neuron, or it is the neuron’s attempt to contain it. The remainder of this study distinguishes between them.

### 3.2 Silencing neuronal transferrin aggravates iron overload and neuronal death

If transferrin supplies the iron that drives ferroptosis, then removing transferrin should reduce injury. It did not; knockdown made every measure of injury worse. It did not. Three weeks after intraventricular AAV-TRF shRNA, total iron in perihaematomal tissue on day 3 after ICH was substantially higher than in shRNA-naive ICH rats (6.3 vs. 4.7 nmol/mg protein; Figure 3A), as were H_2_O_2_ and MDA (Figures 3C,E). Empty-vector controls were indistinguishable from untreated controls throughout, and AAV itself had no effect in the absence of haemorrhage. Ferrous iron and LPO increased with ICH but were not pushed higher by knockdown (Figures 3B,D). This has a straightforward explanation. After a bleed, the neuronal labile iron pool is already close to saturation, so a further loss of buffering capacity shows up mainly as more total iron and more of the oxidative damage that unbuffered iron produces rather than as a further rise in the ferrous reading itself. On this reading, the endogenous transferrin response, a mere threefold rise, is spent by day 3, and removing it exposes how little reserve was left. The structural consequences followed biochemistry. Mitochondria in perihaematomal neurons, already shrunken after ICH, shrank further under knockdown, with fewer cristae and more vacuoles (0.28 vs. 0.60 μm^2^; Figures 3F,G). FJC-positive degenerating neurons increased from 31% to 47% (Figures 3H,I) and lesion volume from 20.1 to 25.3 mm^3^ (Figures 3J,K). Depriving neurons of transferrin made every measure of haemorrhagic injury worse.

**FIGURE 3:**
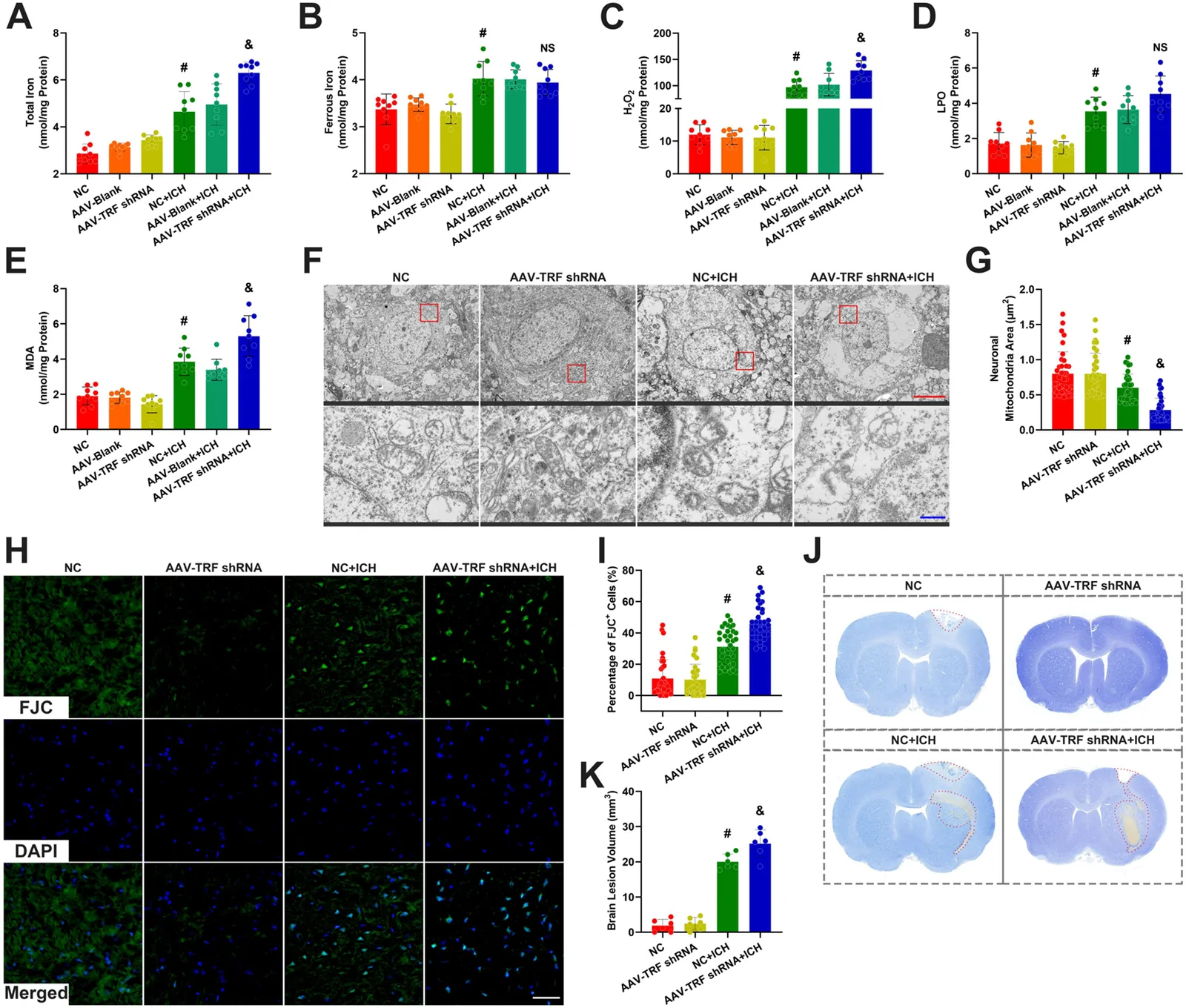
TRF deficiency exacerbates neuronal ferroptosis and aggravates acute outcomes of rats after ICH: **(A–E)** Quantification of total iron, ferrous iron, H_2_O_2_, LPO, and MDA, respectively. N = 9. **(F)** Representative images of TEM of neuronal mitochondria. Upper image, the global cell morphology; lower image, the magnified morphology of the mitochondria of interest outlined by the red box in the upper image. Red scale bar = 5 μm; blue scale bar = 500 nm. **(G)** Quantification of the mitochondrial area. N = 3. **(H)** Representative images of FJC staining for neurodegeneration. DAPI, marker of the cellular nucleus; FJC, marker of degenerative neurons. Scale bar = 50 μm. **(I)** Quantification of FJC-positive cells. N = 3. **(J)** Representative images of Nissl staining for brain lesions (red dashed line enclosed). **(K)** Quantification of brain lesion volume. N = 6. NC, group of normal control rats subjected to vehicle administration; AAV-Blank, group of normal control rats subjected to blank AAV administration; AAV-TRF shRNA, group of normal control rats subjected to AAV carrying TRF shRNA administration; NC + ICH, group of NC subjected to ICH operation; AAV-Blank + ICH, group of AAV-Blank subjected to ICH operation; AAV-TRF shRNA + ICH, group of AAV-TRF shRNA subjected to ICH operation. #, *p* < 0.05 versus NC; &, *p* < 0.05 versus NC + ICH; NS, no significance versus NC + ICH. All data were expressed as the mean ± SD.

### 3.3 Raising neuronal transferrin drives iron below baseline and protects the brain

The converse manipulation produced the mirror image and did so with a margin that a delivery model cannot accommodate. In AAV-TRF rats, ICH failed to raise total iron at all: on day 3, perihaematomal total iron was 3.9 nmol/mg protein against 7.1 in control ICH rats and 5.1 in uninjured controls (Figure 4A). Ferrous iron behaved the same way, falling to 3.0 against 5.1 after ICH and 3.8 at baseline (Figure 4B). Overexpressing transferrin left the tissue with less iron than an uninjured brain. This is the behaviour of a sink, not a supply line. H_2_O_2_, LPO, and MDA were all reduced accordingly (Figures 4C–E); the neuronal mitochondrial area was restored from 0.56 to 0.81 μm^2^ with visibly preserved cristae (Figures 4F,G); FJC-positive neurons fell from 31% to 20% (Figures 4H,I); and lesion volume fell from 22.1 to 14.5 mm^3^ (Figures 4J,K). Taken with Section 3.2, transferrin is bidirectionally rate-limiting for neuronal survival after ICH, and its sign is the opposite of the one the canonical model assigns it.

**FIGURE 4:**
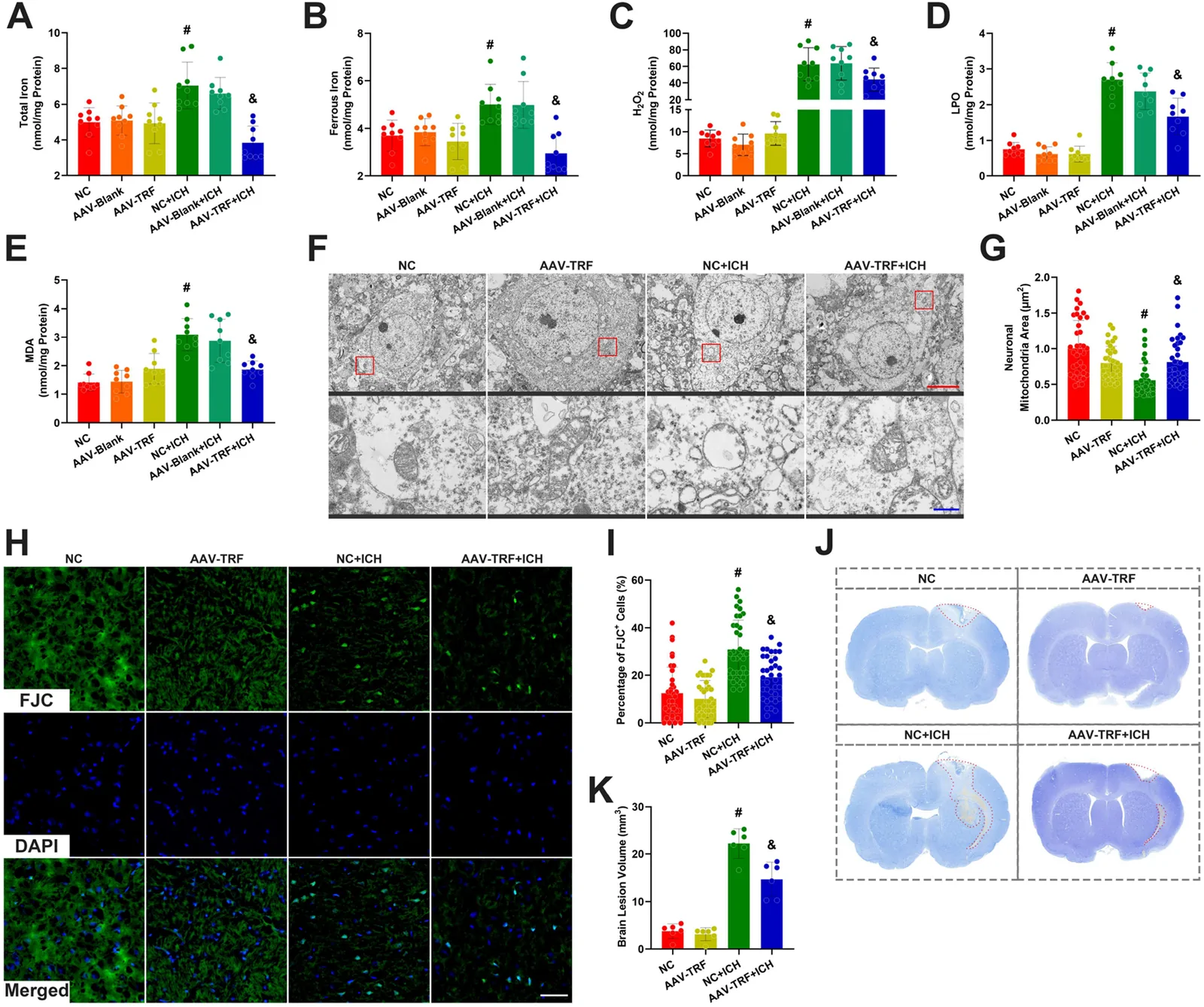
TRF overexpression alleviates neuronal ferroptosis and ameliorates acute outcomes of rats after ICH: **(A–E)** Quantification of total iron, ferrous iron, H_2_O_2_, LPO, and MDA, respectively. N = 9. **(F)** Representative images of TEM for neuronal mitochondria. Upper image, global cell morphology; lower image, the magnified morphology of the mitochondria of interest outlined by the red box in the upper image. Red scale bar = 5 μm; blue scale bar = 500 nm. **(G)** Quantification of the mitochondrial area. N = 3. **(H)** Representative images of FJC staining for neurodegeneration. DAPI, marker of the cellular nucleus; FJC, marker of degenerative neurons. Scale bar = 50 μm. **(I)** Quantification of FJC-positive cells. N = 3. **(J)** Representative images of Nissl staining for brain lesions (red dashed line enclosed). **(K)** Quantification of brain lesion volume. N = 6. NC, group of normal control rats subjected to vehicle administration; AAV-Blank, group of normal control rats subjected to blank AAV administration; AAV-TRF, group of normal control rats subjected to AAV carrying TRF gene administration; NC + ICH, group of NC subjected to ICH operation; AAV-Blank + ICH, group of AAV-Blank subjected to ICH operation; AAV-TRF + ICH, group of AAV-TRF subjected to ICH operation. #, *p* < 0.05 versus NC; &, *p* < 0.05 versus NC + ICH. All data were expressed as the mean ± SD.

### 3.4 Inversion is intrinsic to the neuron

Because the intact brain contains microglia, astrocytes, plasma-derived transferrin, and a blood–brain barrier of uncertain integrity, we repeated both manipulations in hemin-exposed PC-12 neurons, where the transferrin under experimental control is the cells' own. Knockdown reproduced the *in vivo* result. Hemin reduced viability to 78%, and TRF-KD cells fell to 50% (Figure 5A), with more total iron, H_2_O_2_, and MDA (Figures 5B–D). Mitochondrial membrane potential, already collapsed by hemin, collapsed further, with fewer red JC-1 aggregates and more green monomers (Figures 5E,F); mitochondria were smaller with sparser cristae (Figures 5G,H); and Annexin V-FITC/PI double-positive cells increased from 22% to 26% (Figures 5I,J). Overexpression again inverted every one of these. Viability under hemin improved from 68% to 80% (Figure 5K), and intracellular total iron, H_2_O_2_, and MDA all fell, H_2_O_2_ to 5.1 μmol/mg protein, indistinguishable from the 5.2 of unexposed wild-type cells (Figures 5L–N). Membrane potential was partly restored (Figures 5O,P), the mitochondrial area recovered (Figures 5Q,R), and apoptosis fell from 21% to 13% (Figures 5S,T). A neuron making more of its own transferrin shows no greater H_2_O_2_ or MDA accumulation than a neuron that was never exposed to hemin. Since transferrin is a secreted protein, the most economical explanation is that it is acting where it is secreted, in the extracellular space, on the iron before it arrives.

**FIGURE 5:**
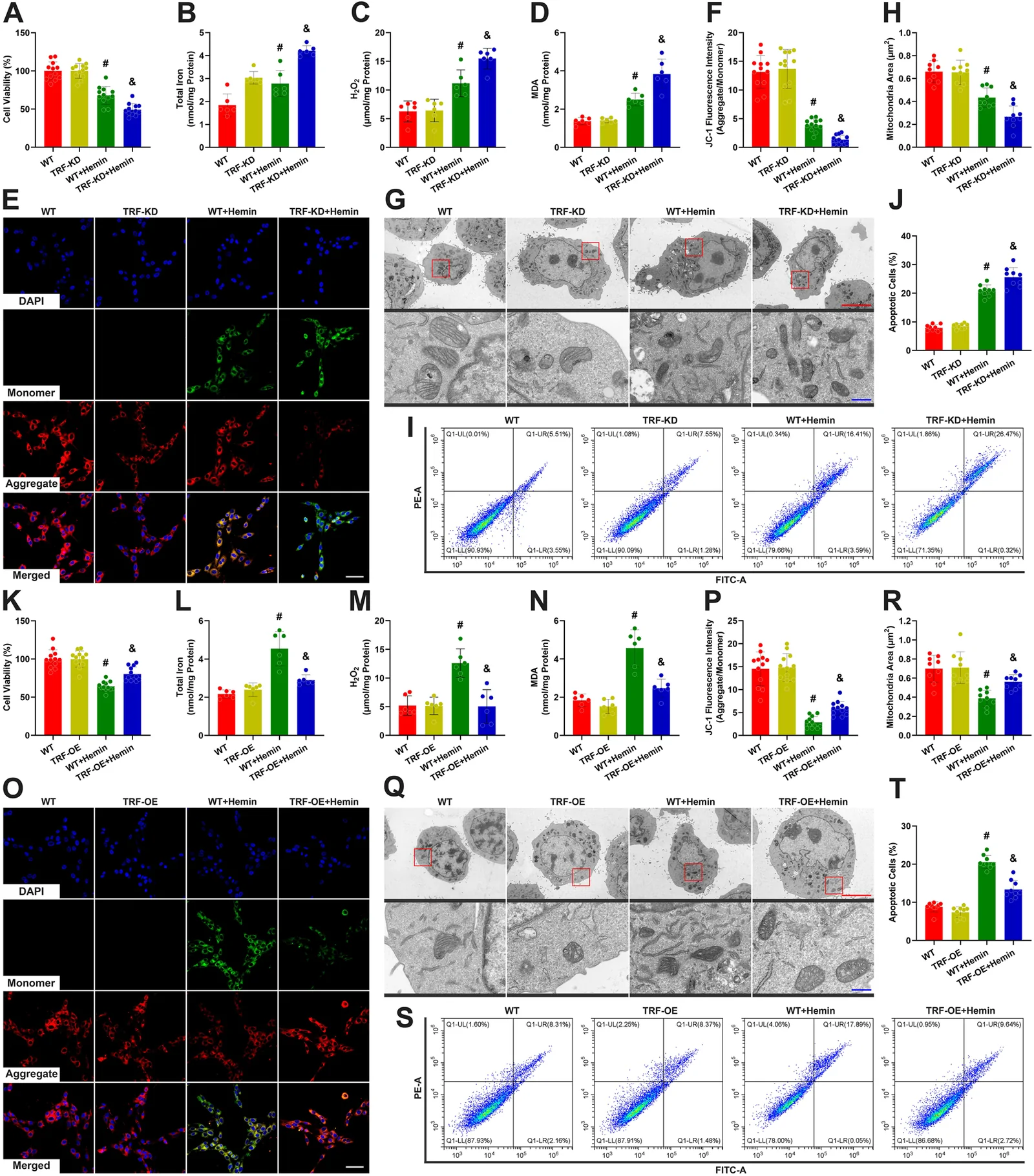
Role of TRF in the ICH-like injury model *in vitro*: **(A,K)** Cell viability. **(B,L)** Quantification of total iron. **(C,M)** Quantification of H_2_O_2_. **(D,N)** Quantification of MDA. **(E,O)** Representative images of JC-1 staining for mitochondrial membrane potential. DAPI, marker of the cellular nucleus. Aggregate, indicator of high membrane potential; monomer, indicator of low membrane potential. Scale bar = 50 μm. **(F,P)** Quantification of JC-1 fluorescence intensity. **(G,Q)** Representative images of TEM for mitochondria. Upper image, global cell morphology; lower image, the magnified morphology of the mitochondria of interest outlined by the red box in the upper image. Red scale bar = 5 μm; blue scale bar = 500 nm. **(H,R)** Quantification of the mitochondrial area. **(I,S)** Representative images of flow cytometry for cell apoptosis. **(J,T)** Quantification of apoptotic cells. For the cell viability assay, there were 12 samples per group; for the measurement of total iron, H_2_O_2_, and MDA levels, there were six samples per group; for JC-1 staining and TEM, there were three samples per group; for flow cytometry, there were nine samples per group. WT, group of wild-type cells subjected to vehicle treatment; TRF-KD, group of wild-type cells subjected to TRF siRNA treatment; TRF-OE, group of wild-type cells subjected to plasmid carrying TRF gene treatment; WT + Hemin, group of WT exposed to Hemin; TRF-KD + Hemin, group of TRF-KD exposed to Hemin; TRF-OE + Hemin, group of TRF-OE exposed to Hemin. #, *p* < 0.05 versus WT; &, *p* < 0.05 versus WT + Hemin. All data were expressed as the mean ± SD.

### 3.5 Transferrin abolishes the injury programme rather than reinforcing antioxidant defence

We then asked what hemin does to a neuron that has enough transferrin. RNA sequencing gave an unexpectedly categorical answer: to the first approximation, it does nothing. In wild-type neurons, hemin changed the expression of 6,976 genes. In neurons overexpressing transferrin, the same exposure changed 343, a 95% collapse of the transcriptional response (Figure 6A; Supplementary Figures S2A,B). Transferrin very largely prevented the response to hemin.

**FIGURE 6:**
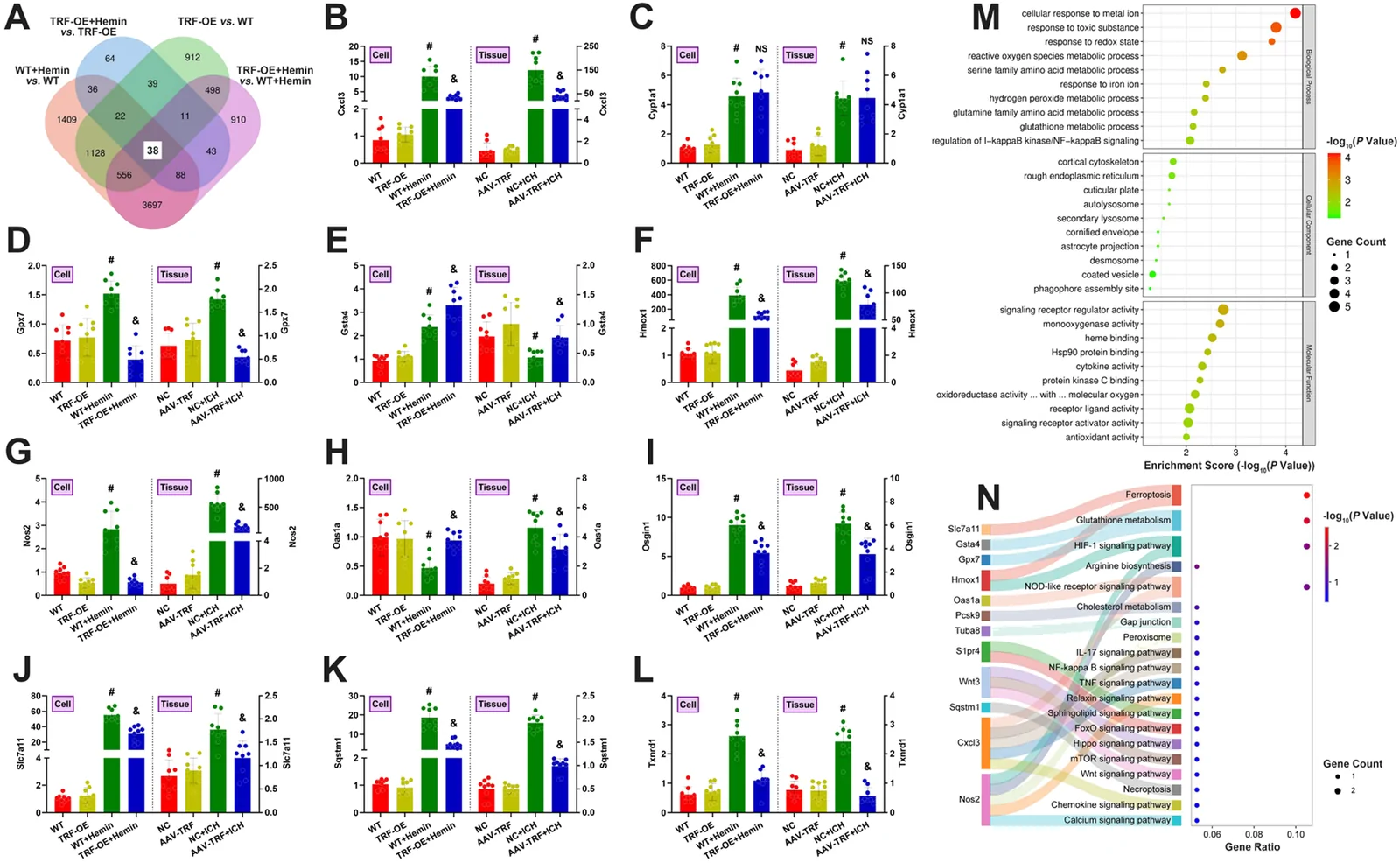
Potential molecular mechanism of TRF exerting cytoprotection in the ICH-like injury *in vitro* model: **(A)** Gene expression characteristics visualised in a Venn diagram. N = 3. **(B-L)** RT-qPCR validation of *Cxcl3, Cyp1a1, Gpx7, Gsta4, Hmox1, Nos2, Oas1a, Osgin1, Slc7a11, Sqstm1*, and *Txnrd1* genes, respectively. N = 9. **(M)** The top 10 most significant enrichment terms in the biological process, cellular component, and molecular function, respectively, of intersected genes in GO analysis. The more genes enriched, the larger the spots; the higher the enrichment score (−log10 scale of P-value), the closer the colour of the spots tended to be red. **(N)** The enrichment terms of intersected genes in KEGG analysis. The more genes enriched, the larger the spots; the higher the enrichment score (−log10 scale of P-value), the closer the colour of the spots tended to be red. WT, group of wild-type cells subjected to vehicle treatment; TRF-OE, group of wild-type cells subjected to plasmid carrying TRF gene treatment; WT + Hemin, group of WT exposed to Hemin; TRF-OE + Hemin, group of TRF-OE exposed to Hemin; NC, group of normal control rats subjected to vehicle administration; AAV-TRF, group of normal control rats subjected to AAV carrying TRF gene administration; NC + ICH, group of NC subjected to ICH operation; AAV-TRF + ICH, group of AAV-TRF subjected to ICH operation. #, *p* < 0.05 versus WT or NC; &, *p* < 0.05 versus WT + Hemin or NC + ICH; NS, no significance versus WT + Hemin or NC + ICH. All data were expressed as the mean ± SD.

Prevention and reversal are different claims, reversal being the transcriptional counterpart of the functional inversion already demonstrated in Sections 3.2–3.4, so we tested the stronger one directly. Throughout, reversal denotes opposition of sign between the two effects, not restoration of expression to the untreated baseline. For each of the 6,974 hemin-responsive genes with data in both contrasts, we compared the change produced by hemin with the change attributable to transferrin in hemin-exposed cells (TRF-OE + Hemin versus WT + Hemin). The two were strongly anticorrelated (Pearson r = −0.869, Spearman ρ = −0.896; Supplementary Figure S1A). Transferrin moved 92.4% of hemin-responsive genes in the direction opposite to hemin, and among the 4,381 genes that both perturbations moved past the significance threshold, 99.0% were reversed. The directional composition mirrors this as follows: hemin acted predominantly by induction (4,875 genes up and 2,101 down), whereas transferrin in the hemin-exposed state acted predominantly by suppression (1,220 up and 4,623 down) (Supplementary Figure S1B; Supplementary Figure S2). Transferrin does not blunt the injury programme along some independent axis. It runs it backwards.

Because those two contrasts share the hemin-exposed wild-type samples, noise in that group alone could in principle manufacture an anticorrelation, so we tested the result in three ways. Estimating the hemin effect from two of the three hemin-exposed replicates and the transferrin effect from the remaining one, leaving the two estimates with no sample in common, returned r = −0.874 (mean of the three rotations, range −0.878 to −0.872; n = 5,505), indistinguishable from the original. Permuting gene labels gave a null centred on 0 (mean r = 0.000, SD 0.013), placing the observed value approximately 67 standard deviations from the null mean; no permutation reached the observed value, giving an empirical P < 0.0005. This is a descriptive comparison with the permutation null rather than a formal significance test. Finally, we compared two contrasts sharing no samples at all: the effect of hemin measured in wild-type cells against the effect of hemin measured in transferrin-overexpressing cells. The median absolute log2 fold change fell from 1.99 to 0.28, an approximately sevenfold reduction in the median absolute log2 fold change and 80.7% of the genes that hemin moved in wild-type cells failed to reach the significance threshold in transferrin-overexpressing cells. The collapse of the injury programme is therefore not an artefact of shared samples, and it is measurable as an effect size rather than only as a gene count.

Thirty-eight were shared across all four comparisons (Figure 6A; Supplementary Table S2). Eleven were re-measured by RT-qPCR in both hemin-exposed cells and day-3 perihaematomal tissue, and the cell and tissue data agreed closely (Figures 6B–L), showing that the *in vitro* programme is the one the injured brain actually runs. Seven of the eleven genes (*Gpx7, Hmox1,* Oas1a, *Osgin1, Slc7a11, Sqstm1,* and *Txnrd1*) also matched the direction called by sequencing. Four did not. *Cxcl3, Cyp1a1, Gsta4,* and *Nos2* were called down after hemin by sequencing but measured up by RT-qPCR in both cells and tissue. We report this discrepancy without resolving it. It cannot be dismissed as sequencing noise: the effects are large and the triplicates tight (for *Nos2,* normalised counts of 7,004, 6,420, and 5,302 in untreated cells against 81, 107, and 148 after hemin), with adjusted *p*-values from 1.3 × 10^−5^ to 2.3 × 10^−67^ and mean normalised counts well above the low-expression range for all four. Nor can it be dismissed as a qPCR artefact on the face of it, since the two systems were measured independently at n = 9 and agreed with each other. What we have are two internally consistent measurements that contradict each other, most plausibly at the level of the assay, primer specificity, or the detection of different transcript isoforms, rather than in biology. We note that the sequencing direction for *Nos2* is at least biologically coherent, since the tenfold induction of *Hmox1* in the same cells would be expected to suppress inducible nitric oxide synthase, but we offer that as a consideration, not a verdict. Confirming the four discordant genes will require independent primers and a third method. No argument in this paper depends on them: the mechanistic account below rests on the seven concordant genes, which happen to be the ones that constitute the NRF2-type programme.

The seven concordant genes tell a coherent story, and it is not the expected one. Hemin drove a large NRF2-type oxidative stress programme: *Hmox1* increased 3.3-fold by sequencing (a rise from ∼1,000 to ∼13,600 TPM, by far the largest single response in the dataset), with *Slc7a11* up 3.4-fold, *Txnrd1* 1.7-fold, *Sqstm1* 1.8-fold, and *Osgin1* 3.1-fold. Transferrin overexpression pushed every one of them back down (Figures 6F,I–L), and the same pattern held in AAV-TRF brains after ICH. Read conventionally, this looks like harm: SLC7A11 sustains glutathione synthesis, TXNRD1 sustains thioredoxin, and suppressing them should sensitise a cell to ferroptosis. Yet the same cells were protected on every functional measure. The contradiction dissolves once these genes are read as an alarm rather than a defence. Their induction reports unbuffered, redox-active iron; transferrin removes the stimulus, so the alarm is never raised. The magnitude of the antioxidant response after ICH is thus a readout of buffer failure, not a driver of survival, and its suppression by transferrin is evident that insult was intercepted upstream. HO-1 makes this concrete. HO-1 degrades heme and, in doing so, liberates the ferrous iron that feeds Fenton chemistry, so a 13-fold induction is not only a marker of heme stress but an amplifier of it. By holding iron at the source, transferrin cut *Hmox1* induction roughly fourfold and broke the heme–HO-1–free iron–more heme stress loop before it could close. Enrichment analysis agreed. The shared genes populated GO terms for cellular response to metal ion, response to redox state, ROS metabolic process, response to iron ion, and hydrogen peroxide metabolic process, acting through monooxygenase, heme-binding, oxidoreductase, and antioxidant activities (Figure 6M); KEGG returned ferroptosis (rno04216) and glutathione metabolism (rno00480) as the two most significant pathways, followed by HIF-1, NOD-like receptor, peroxisome, NF-κB, and necroptosis signalling (Figure 6N; Supplementary Tables S3 and S4). The programme transferrin switches off is, by unbiased annotation, the iron-and-redox programme of haemorrhagic injury.

### 3.6 Transferrin reverses a coordinated failure of iron handling

The genome-wide analysis shows that transferrin reverses the hemin injury programme, but it does not on its own speak to the iron-handling machinery that our central claim is about. We therefore defined, before inspecting the results, a panel of iron-handling and ferroptosis effector genes covering the transferrin–TfR1–DMT1 uptake pathway, cytosolic iron sensing, ferritin storage, the hepcidin–ferroportin export axis, non-transferrin-bound iron (NTBI) transport, heme handling, and the lipid peroxidation and cyst(e)ine-glutathione effectors of ferroptosis and report it here in Figure 7 (Supplementary Table S5).

**FIGURE 7:**
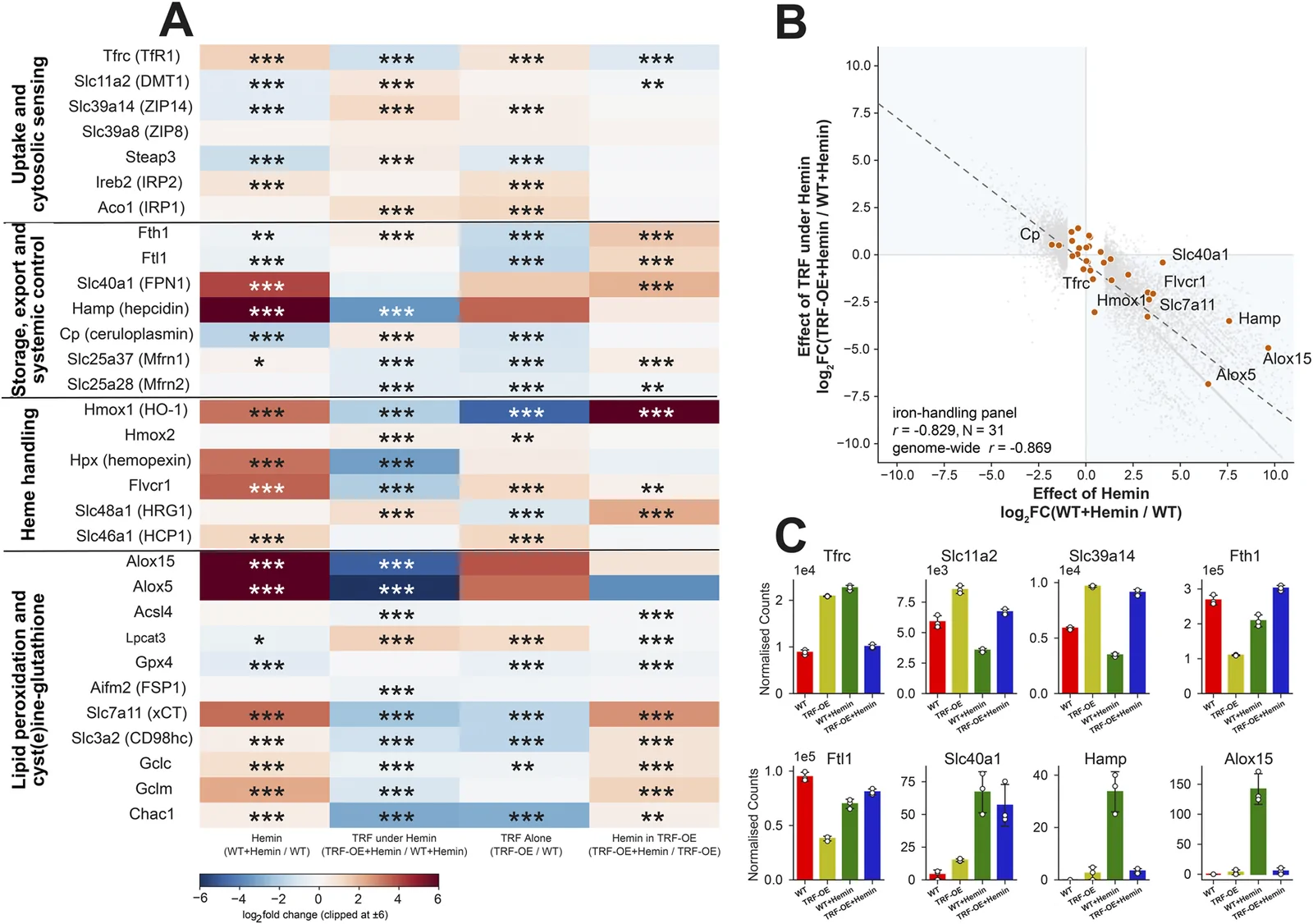
A pre‐specified panel of iron‐handling and ferroptosis‐effector genes shows that transferrin reverses a coordinated failure of iron handling under hemin exposure. **(A)** Heatmap of log2 fold change (clipped at ±6) for 31 genes spanning four functional modules (uptake and cytosolic sensing; storage, export and systemic control; heme handling; lipid peroxidation and cyst(e)ine‐glutathione), across four comparisons: the effect of hemin in wild‐type cells (Hemin, (WT + Hemin)/WT), the effect of transferrin overexpression in hemin‐exposed cells (TRF under Hemin, (TRF‐OE+Hemin)/(WT + Hemin)), the effect of transferrin overexpression alone (TRF Alone, TRF‐OE/WT), and the effect of hemin in transferrin‐overexpressing cells (Hemin in TRF‐OE, (TRF‐OE + Hemin/TRF‐OE). *, P < 0.05; **, P < 0.01; ***, P < 0.001 (adjusted). **(B)** Correlation between the effect of hemin and the effect of transferrin under hemin, for the iron‐handling panel (orange points; Pearson r = −0.829, N = 31) overlaid on the genome‐wide relationship (grey points; r = −0.869, as in Figure 6). Selected genes are labelled. **(C)** Normalised counts for eight representative genes (*Tfrc, Slc11a2, Slc39a14, Fth1, Ftl1, Slc40a1, Hamp, Alox15*) across four groups. N = 3. WT, group of wild type cells subjected to vehicle treatment; TRF‐OE, group of wild type cells subjected to plasmid carrying TRF gene treatment; WT+Hemin, group of WT exposed to Hemin; TRF‐OE + Hemin, group of TRF‐OE exposed to Hemin. All data were expressed as mean ± SD.

Hemin did not simply raise iron inside the neuron; it left the cell able to import iron but unable to store or export it. Import was favoured: *Tfrc,* encoding TfR1, increased (log_2_ fold change + 1.36), and Steap3, the endosomal ferrireductase that converts Fe^3+^ to Fe^2+^, fell (−1.43). Storage moved the other way, with Fth1 and Ftl1 both falling (−0.36 and −0.44) instead of rising as an iron-loaded cell should. Export was engaged but neutralised: Slc40a1 (ferroportin) was induced (+4.07), yet Hamp (hepcidin), whose product internalises and degrades ferroportin, increased far more (+7.58); Cp (ceruloplasmin), required for the ferroxidase step that permits iron to leave the cell, fell (−1.80). Slc39a14, the principal transporter for non-transferrin-bound iron, fell (−0.76). Transferrin reversed this configuration gene by gene: *Tfrc* fell by almost exactly the amount hemin had raised it (−1.35), Steap3 increased (+0.50), Fth1 and Ftl1 returned towards baseline (+0.35 and +0.03), Hamp fell (−3.51), Cp increased (+0.53), and Slc39a14 increased (+1.21).

These are transcript-level measurements from a targeted but prespecified panel, not protein-level or functional measurements: TfR1, DMT1, ferritin, ferroportin, hepcidin, and ZIP14 proteins were not measured in tissue or in cerebrospinal fluid and plasma, and transcript changes, particularly for ferroportin and ZIP14, which are heavily regulated post-translationally, should not be assumed to report protein abundance directly. What this analysis establishes is narrower but useful: the iron-handling machinery is not a blind spot in this dataset, and transferrin’s effect on it is coherent and moves in the direction the buffering hypothesis predicts.

### 3.7 Recombinant apo-transferrin is therapeutic, with a threshold dose and lasting functional benefit

Genetic manipulation establishes the mechanism but cannot be given to patients. We therefore gave humanised recombinant apo-transferrin intraperitoneally, starting 1 h after the bleed. The dose response was the most mechanistically revealing result of the study. At 4 and 10 mg/kg, hTRF did nothing measurable to lesion volume (24.8 and 21.0 mm^3^ against 22.9 for vehicle, both non-significant). At 20 mg/kg, it more than halved it, to 10.2 mm^3^ (Figures 8A,B). Pharmacological agents acting through a receptor or a signalling pathway generally titrate; a buffer does not. It does nothing until its capacity exceeds the load it must absorb, and then, it works. The step between 10 and 20 mg/kg is what a stoichiometric iron sink looks like, and it is difficult to reconcile with any mechanism in which transferrin acts as a ligand. At 20 mg/kg, biochemistry echoed the AAV data with one instructive difference. Total iron fell only modestly, from 9.1 to 7.8 nmol/mg protein, still above the 7.3 of Sham (Figure 8C), and ferrous iron from 6.7 to 5.3 (Figure 8D). Yet H_2_O_2_ fell from 220 to 7 nmol/mg protein, below the Sham value of 19 (Figure 8E), LPO from 2.2 to 0.75 (Figure 8F), and MDA from 4.0 to 2.1 (Figure 8G). A 14% fall in total iron accompanied by a 30-fold fall in H_2_O_2_ cannot be explained by iron removal. It is explained by iron speciation: apo-transferrin did not clear the iron from the tissue; it took it out of circulation chemically, and iron that is bound at Kd ∼10^−22^ M is iron that cannot run Fenton chemistry no matter how much of it is present. This is the pharmacological signature of buffering rather than chelation, and it is what allows a protein to disarm an iron load without stripping the tissue of iron. The structural and functional consequences were substantial. The neuronal mitochondrial area recovered from 0.49 to 0.69 μm^2^ (Figures 8H,I), and FJC-positive degenerating neurons fell from 53% to 13%, close to the 8% of Sham (Figures 8J,K). Sensorimotor deficits on day 3 were reversed on both the corner-turning and forelimb-placing tests (Figures 8L,M). At day 14, eleven days after the last measurement of iron chemistry—treated rats searched the maze in structured paths rather than drifting (Figure 8N), reached the target quadrant in 61 s against 114 s for vehicle (Figure 8O), and crossed the platform site more often (Figure 8P). A 3-day course of an iron-binding protein, begun an hour after the bleed, was still associated with preserved cognition 2 weeks later.

**FIGURE 8:**
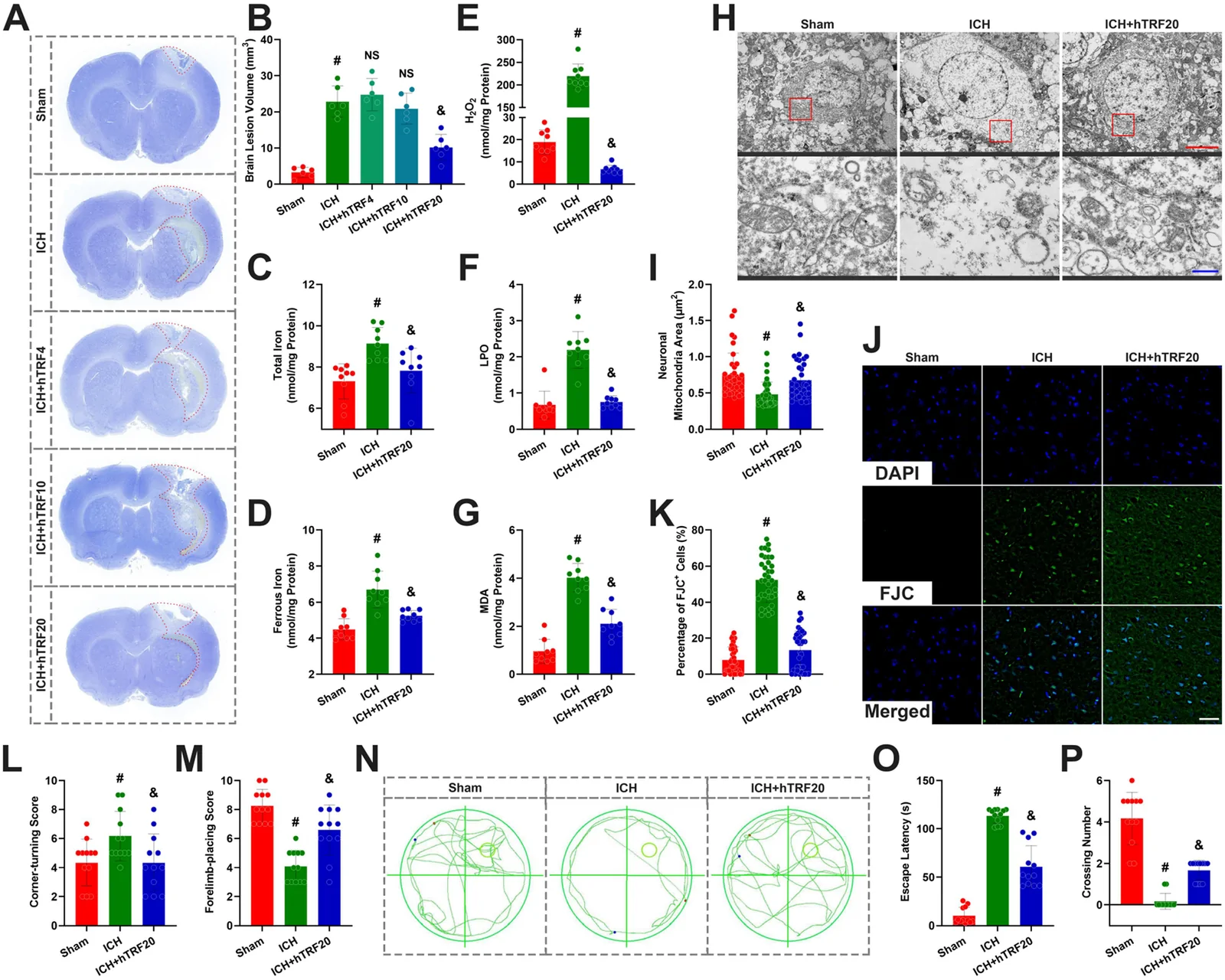
Administration of exogenous TRF inhibits neuronal ferroptosis and improves functional outcomes of rats after ICH: **(A)** Representative images of Nissl staining for brain lesions (red dashed line enclosed). **(B)** Quantification of brain lesion volume. N = 6. **(C–G)** Quantification of total iron, ferrous iron, H_2_O_2_, LPO, and MDA, respectively. N = 9. **(H)** Representative images of TEM for neuronal mitochondria. Upper image, the global cell morphology; lower image, the magnified morphology of the mitochondria of interest outlined by the red box in the upper image. Red scale bar = 5 μm; blue scale bar = 500 nm. **(I)** Quantification of the mitochondrial area. N = 3. **(J)** Representative images of FJC staining for neurodegeneration. DAPI, marker of the cellular nucleus; FJC, marker of degenerative neurons. Scale bar = 50 μm. **(K)** Quantification of FJC-positive cells. N = 3. **(L)** Corner-turning score in the corner-turning test. N = 12. **(M)** Forelimb-placing score in the forelimb-placing test. N = 12. **(N)** Representative swimming path in the MWM test. **(O)** Escape latency in the MWM test. N = 12. **(P)** Crossing number in the MWM test. N = 12. Sham, group subjected to sham operation and vehicle administration; ICH, group subjected to ICH operation and vehicle administration; ICH + hTRF4, group subjected to ICH operation and 4 mg/kg hTRF administration; ICH + hTRF10, group subjected to ICH operation and 10 mg/kg hTRF administration; ICH + hTRF20, group subjected to ICH operation and 20 mg/kg hTRF administration. #, *p* < 0.05 versus Sham; &, *p* < 0.05 versus ICH; NS, no significance versus ICH. All data were expressed as the mean ± SD.

## 4 Discussion

The transferrin system is among the best-understood in cell biology, and its established function is the delivery of iron. TfR1 is a ferroptosis marker because ferroptosing cells import transferrin-bound iron (Feng et al., 2020); blocking that import protects cells from ferroptotic death (Feng et al., 2020; Xiong et al., 2014). Our results run against this in every particular. Knocking transferrin down in neurons increased perihaematomal iron and killed more of them, raising it drove iron below the level of an uninjured brain and halved neurodegeneration, and giving the protein as a drug after the bleed rescued lesion volume and cognition. The direction of every effect is the opposite of what the canonical model predicts.

We do not think the canonical model is wrong; we think that it has been extended past its domain. It describes a cell whose iron arrives as diferric transferrin at a receptor, under conditions in which delivery is the regulated step. A haemorrhagic brain is not that system. Iron arrives as heme and as non-transferrin-bound species, in quantities set by the size of the clot rather than by any receptor, and the regulated step is not delivery but containment. In that context, the relevant property of transferrin is the ∼70% of its iron-binding sites that stand empty under physiological saturation (Gaasch et al., 2007). Transferrin stops functioning as a vehicle and starts functioning as a sink. Its sign flips because its substrate changed, not because its biochemistry did.

Four features of the data are difficult to explain in any other way. The first is the dose threshold: 4 and 10 mg/kg of hTRF were inert, and 20 mg/kg halved the lesion. Ligands titrate; buffers have capacities. The second is the dissociation between iron mass and iron chemistry: hTRF lowered total iron by 14% while lowering H_2_O_2_ thirtyfold. No amount of iron removal explains that, but sequestration does, because a transferrin-bound ferric ion is chemically silent regardless of how much of it is there. The third is that overexpression drove tissue iron below the uninjured baseline, which a delivery system cannot do in the direction observed.

The fourth is the shape of the transcriptional response, which also tells us where in the cascade transferrin acts. Hemin moved 6,976 genes in wild-type neurons and 343 in transferrin-overexpressing ones. That is not the profile of a cell coping well with an insult; it is the profile of a cell that never received it. The residual response amounts to the injury programme running in reverse: across all 6,974 hemin-responsive genes, the two effects were anticorrelated at r = −0.869, with 99.0% of the genes that both perturbations moved significantly running in opposite directions. A protein acting downstream (reinforcing a defence, accelerating a repair pathway, and buffering a signal) would leave the injury programme visible and add its own. Only an agent that removes the initiating stimulus can return several thousand genes towards where they started. The corollary is the finding that looks most like a contradiction and is the strongest evidence: transferrin suppressed the NRF2-type programme (*Hmox1, Slc7a11, Txnrd1, Sqstm1,* and *Osgin1*) while protecting the cell. If one assumes that these genes are the mechanism of survival, the result is incoherent. If one recognises that they are the cell’s alarm, raised in proportion to unbuffered iron, the result is exactly what upstream interception predicts. Suppressing them costs nothing, because there is no longer anything for them to defend against.

This reconciles our work with the one previous report of exogenous apo-transferrin in ICH: the protein improved neurobehaviour in mice without moving classical ferroptosis markers or reducing haemorrhage volume, and the authors concluded that the benefit was related to iron metabolic pathways rather than the GSH/GPX4 system (García-Serran et al., 2023). We reach the same conclusion from the opposite direction and can now say why. The GSH/GPX4 axis is not engaged because TRF acts before the point at which that axis would be needed. That study’s failure to reduce haemorrhage volume also acquires an explanation. Its dosing sat where our own dose response is flat; below the buffering threshold, an iron sink is simply an inert protein. The distinction between an ineffective drug and an underdosed one is not academic here, and our data argue for the latter.

The endogenous response is the other half of the story, and its most striking feature is its inadequacy. The brain mounts an appropriate but insufficient response after a bleed. It makes more transferrin, in the two cell types that handle heme iron, peaking precisely when ferroptosis peaks, and the response is far too small. A threefold rise cannot buffer a 100 μL bolus of blood, which is why the knockdown data show so little ferrous headroom left to lose (Figure 3B) and why AAV-mediated overexpression and 20 mg/kg of exogenous protein have anything to add at all. The therapeutic opportunity here is not the discovery of a new target but the recognition that an existing, correct, endogenous response saturates well below what the injury demands and can be topped up. This also explains the clinical observation that motivated the study: transferrin is higher in ICH patients with smaller haematomas and better outcomes (Yang et al., 2016), our data suggest an interpretation of this association - that the patients whose buffering reserve was least overwhelmed did best - although the clinical observation is associative and our experiments cannot establish causation in patients.

Framed this way, transferrin occupies a different position from the chelators the field has already tested. Deferoxamine failed to meet its primary endpoint in i-DEF (Selim et al., 2019), and its difficulties, such as indiscriminate stripping of iron from metalloenzymes, poor brain penetration, and dose-limiting toxicity, follow from what a chelator is. Transferrin is not a chelator. It is endogenous to the brain, already upregulated by the injury, binds ferric iron with an affinity that exceeds deferoxamine, releases it only in acidified endosomes, and leaves the iron in the tissue while rendering it inert. Apo-transferrin has been administered to humans as plasma-derived apotransferrin in congenital atransferrinaemia, so the protein class is not new to clinical use; this may ease, but does not by itself establish, a translational path for the indication studied here. Apo- and holo-transferrin is not interchangeable: iron-loaded transferrin increases ROS and injures neurons deprived of oxygen and glucose, whereas the iron-free form protects (DeGregorio-Rocasolano et al., 2018). Apo-transferrin also protects cortical neurons against haemoglobin, with a potency comparable with deferoxamine at equivalent iron-binding capacity, and this effect is not antagonised by holo-transferrin (Chen-Roetling et al., 2011). Everything in our data is consistent with capacity (empty binding sites) being the active principle. More broadly, our finding that transferrin suppresses the SLC7A11-centred NRF2 module sits within a wider framework of therapeutic modulation of metal dyshomeostasis, which spans site-specific iron and copper chelation strategies for neuroprotection and the recently described role of SLC7A11 as a node connecting ferroptosis to disulfidptosis (Tolbatov and Marrone, 2026a; Tolbatov and Marrone, 2026b). The suppression of *Slc7a11* and its NRF2-module partners in transferrin-overexpressing neurons is consistent with a reduced need for antioxidant defence once the initiating iron stimulus is intercepted, rather than the loss of a protective pathway.

Our study has real limitations. We infer buffering from total and ferrous iron, from the dose threshold and from the mass–chemistry dissociation, but we did not measure the labile iron pool, non-transferrin-bound iron, or transferrin saturation directly, and those measurements are what would convert a well-supported inference into a demonstration. The iron-handling panel reported in Figure 7 (Results) speaks to several of these open questions, though at the transcript level only. Hemin left the neuron able to import iron but unable to store or export it, and transferrin reversed each step (see Results for the full configuration and specific values); we did not extend this to protein-level or functional measurements, in tissue or in cerebrospinal fluid and plasma, and transcript changes, particularly for ferroportin and ZIP14, which are heavily regulated post-translationally, should not be assumed to report protein abundance directly. Nor does this local, cell-autonomous analysis address the systemic hepcidin–ferroportin axis that regulates whole-body iron flux over a longer timescale (Nemeth et al., 2004); how the two levels of regulation interact after ICH is an open question. What the analysis does establish is that the iron-handling machinery is not a blind spot in this dataset and that transferrin’s effect on it is coherent and in the direction the buffering hypothesis predicts.

Brain transferrin is synthesised principally by choroid plexus epithelium and by oligodendrocytes, not by astrocytes (Bloch et al., 1985; Jeong and David, 2003); astrocytes instead export iron via ferroportin and GPI-anchored ceruloplasmin into an extracellular space where apo-transferrin is already present. The absence of the TRF signal in GFAP-positive cells in Figure 2D is consistent with this division of labour rather than evidence against apo-transferrin availability, and we do not have direct evidence for or against an astrocyte-to-neuron recycling step; we do not claim one here. We also did not stain for oligodendrocytes, which are, together with the choroid plexus, the other principal source of brain transferrin and, in recent single-cell and spatial transcriptomic work, the cell type in which posthaemorrhagic ferroptosis appears most concentrated (Gu et al., 2024); this is a genuine gap in the cell-type coverage of our histology.

Transferrin overexpression is not transcriptionally silent. In unexposed cells, it changed 3,206 genes relative to wild type (Supplementary Figure S2C), so the protected state we characterise is not simply the naive state with more transferrin in it, and some of the protection may derive from that altered baseline rather than from iron binding during the insult. The reversal analysis survives a split-sample control, a permutation null, and a comparison between contrasts sharing no samples, but it remains a correlation: it establishes that the transferrin effect is antiparallel to the hemin effect not that iron sequestration is what makes it so. Four of the eleven validated genes disagreed with the sequencing calls, and since the sequencing for those genes is robust, the discrepancy remains genuinely open, not merely set aside.

PC-12 cells are a neuronal line, not primary neurons, and do not reproduce interactions within the neurovascular unit; the concordance between our cell and tissue RT-qPCR data and the fact that our principal genetic manipulations were performed in the intact brain rather than in PC-12 cells alone mitigate but do not remove this concern. We studied only male rats to reduce iron-handling and injury-response variability associated with the oestrous cycle while establishing the direction and magnitude of the transferrin effect and used a single species and a single ICH model; autologous blood reproduces the mass effect and the iron load but not vessel rupture. Because we did not include female or aged animals and followed animals for only 14 days, our findings do not establish whether hTRF provides lasting protection against delayed neurodegeneration or whether the effect generalises across sex and age; establishing both is necessary before any translational claim can be made. Our neurobehavioural assessment was also limited to sensorimotor testing at day 3 and cognitive testing at day 14; a broader and longer battery, extending beyond 14 days and including additional cognitive and affective domains, is needed to establish whether the functional benefit of transferrin is durable. Our group comparisons were analysed by one-way ANOVA across all groups, although several experiments have a two-factor structure (genotype or treatment × injury); the conclusions rest on differences large enough that a factorial analysis would be unlikely to overturn them, but interaction terms were not formally tested. We did not run deferoxamine as an active comparator, which is the experiment that would show whether buffering is a genuine improvement on chelation or simply a different way of achieving the same thing. The microglial arm remains open: transferrin increased in microglia too, *Hmox1* and *Cxcl3* point towards a microglial inflammatory component, and whether transferrin restrains microglial ferroptosis and the neuroinflammation that follows is untested here (McCarthy et al., 2018; Liu et al., 2022).

Finally, the exogenous protein needs work before it could be given to patients. We tested a single, early dosing window, 1 h after haemorrhage onset, which is earlier than most patients can realistically be treated; whether apo-transferrin remains effective when treatment starts several hours later is untested and must be established before any translational claim is made. We also did not measure hTRF concentration in brain tissue, cerebrospinal fluid, or perihaematomal fluid after intraperitoneal injection; direct evidence of blood-brain barrier penetration is therefore lacking. We do not propose that intact hTRF crosses an intact barrier; the most likely route is access to the perihaematomal compartment through the barrier disruption caused by the haemorrhage, with a possible indirect contribution from buffering in the peripheral circulation. Distinguishing these requires a pharmacokinetic study, which we did not perform and the biochemical and transcriptional changes we observed, although consistent with the protein reaching its site of action, do not substitute for a pharmacokinetic measurement. Half-life, brain penetration across a barrier of variable integrity, and behaviour in the anticoagulated or hypertensive patient all require further study. What our data establish is narrower and, we think, more useful than a drug candidate: after haemorrhage, the brain’s own iron-carrier changes sides, and how much of it there is decides whether neurons live.

## 5 Conclusion

Transferrin restrains, rather than drives, neuronal ferroptosis after intracerebral haemorrhage. Bidirectional manipulation in the intact brain and in isolated neurons shows that its availability is rate-limiting for iron overload, lipid peroxidation, mitochondrial integrity, lesion size, and neurological recovery, with every effect running opposite to the direction predicted by the transferrin-as-iron-donor model. The transcriptional data place its action upstream of the antioxidant response rather than within it: sufficient transferrin reduces the hemin programme by 95% and inverts what remains, reversing 99% of the genes that both perturbations move. The threshold dose response of the recombinant protein, together with its ability to suppress iron chemistry without removing iron mass, identifies buffering capacity as the active variable. The endogenous response is appropriate but saturates far below what the injury requires, and raising it, genetically or with apo-transferrin, protects the brain and preserves cognition. Iron after haemorrhage may not need to be removed so much as held still.

## Data Availability

The raw data supporting the conclusions of this article will be made available by the authors, without undue reservation.
