# Supplementary material for: Transferrin restrains rather than drives neuronal ferroptosis after intracerebral haemorrhage
Source: Front Pharmacol. 2026 Oct 28;17:1945504. doi: 10.3389/fphar.2026.1945504 (PMC13752033; doi:10.3389/fphar.2026.1945504)
Supplement: Supplementary file 1 [file DataSheet1.zip › Supplementary Material/Supplementary Material 1.docx]

**Table S1. Primer sequence.**

| **Gene** | **Primer Sequence (5'→3')** | |
| --- | --- | --- |
| rat_β-Actin | Forward | TGCTATGTTGCCCTAGACTTCG |
|  | Reverse | GTTGGCATAGAGGTCTTTACGG |
| rat_Cxcl3 | Forward | AACCTTCAGGGACTGTTGTGGC |
|  | Reverse | TGGCTATGACTTCTGTCTGGGTG |
| rat_Cyp1a1 | Forward | ACATCCGGGACATCACAGACA |
|  | Reverse | CCCAGCTCCAAAGAGGTCAA |
| rat_Gpx7 | Forward | GGTTTCCCTGGTGGTGAATGTAG |
|  | Reverse | AAGTTTGGGTTAGGTATTTGAAGGC |
| rat_Gsta4 | Forward | AAATAGACGGGATGCTACTGACA |
|  | Reverse | CTTCTTTTTCCTGAGGGGCTTT |
| rat_Hmox1 | Forward | GCTCTATCGTGCTCGCATGAAC |
|  | Reverse | TCTGTGAGGGACTCTGGTCTTTG |
| rat_Nos2 | Forward | TTTCACGACACCCTTCACCAC |
|  | Reverse | TCTGGGTCCTCTGGTCAAACTC |
| rat_Oas1a | Forward | AGGAGGTGGAGTTTGATGTGCT |
|  | Reverse | CATTCGGAGATGAGGATGGTGTA |
| rat_Osgin1 | Forward | AAGGACTGGATGCGGAAGAAA |
|  | Reverse | GCAGTGCTCAGACTTAGCCCATT |
| rat_Slc7a11 | Forward | TATGCTGAATTGGGTACGAGC |
|  | Reverse | TATTACCAGCAGTTCCACCCA |
| rat_Sqstm1 | Forward | TGTGGTGGGAACTCGCTATAAG |
|  | Reverse | AGTGCCCATGTTTCAGCTTCC |
| rat_Trf | Forward | CGCAAGCCAGTGGATCAGTAT |
|  | Reverse | TTAAACAGCAGGTCTTTCCCAA |
| rat_Txnrd1 | Forward | GCTCTGAAAGACTCACGCAACT |
|  | Reverse | TTCCCGTAAGCATTCTCATAGACA |

**Table S2. Intersection genes of differential expression.**

| **Gene ID** | **Gene Symbol** | **Chromosome** | **Start** | **End** | **Strand** | **WT** | | | **TRF-OE** | | | **WT+Hemin** | | | **TRF-OE+Hemin** | | |
| --- | --- | --- | --- | --- | --- | --- | --- | --- | --- | --- | --- | --- | --- | --- | --- | --- | --- |
|  |  |  |  |  |  | **TPM-1** | **TPM-2** | **TPM-3** | **TPM-4** | **TPM-5** | **TPM-6** | **TPM-7** | **TPM-8** | **TPM-9** | **TPM-10** | **TPM-11** | **TPM-12** |
| ENSRNOG00000028043 | Cxcl3 | 14 | 1.7E+07 | 1.7E+07 | - | 10.01 | 9.94 | 11.04 | 5.91 | 6.38 | 6.95 | 3.24 | 4.95 | 7.24 | 11.75 | 14.91 | 14.01 |
| ENSRNOG00000019500 | Cyp1a1 | 8 | 5.8E+07 | 5.8E+07 | + | 3.39 | 2.34 | 2.46 | 1.68 | 2.44 | 1.99 | 0.48 | 0.56 | 0.44 | 5.46 | 5.60 | 5.87 |
| ENSRNOG00000033202 | Fbxo41 | 4 | 1.2E+08 | 1.2E+08 | - | 0.65 | 0.52 | 0.74 | 0.23 | 0.21 | 0.27 | 0.35 | 0.33 | 0.36 | 0.08 | 0.08 | 0.10 |
| ENSRNOG00000030449 | Gsta4 | 8 | 7.9E+07 | 7.9E+07 | + | 73.96 | 77.53 | 84.68 | 36.56 | 37.17 | 39.72 | 30.50 | 28.51 | 27.36 | 75.33 | 73.88 | 70.44 |
| ENSRNOG00000057443 | Nos2 | 10 | 6.4E+07 | 6.4E+07 | + | 43.45 | 36.58 | 28.12 | 24.65 | 22.79 | 24.98 | 0.63 | 0.86 | 1.17 | 11.76 | 10.90 | 11.83 |
| ENSRNOG00000001369 | Oas1a | 12 | 3.6E+07 | 3.6E+07 | + | 0.46 | 0.16 | 0.24 | 1.91 | 2.55 | 2.20 | 0.10 | 0.09 | 0.07 | 0.89 | 0.46 | 0.99 |
| ENSRNOG00000066264 | Pilrb2l2 | 12 | 1.9E+07 | 1.9E+07 | - | 5.60 | 6.41 | 6.48 | 2.55 | 2.77 | 2.72 | 1.97 | 2.05 | 2.91 | 5.85 | 5.96 | 5.60 |
| ENSRNOG00000005302 | Slc2a9 | 14 | 7.2E+07 | 7.2E+07 | + | 0.50 | 0.58 | 0.43 | 0.36 | 0.30 | 0.30 | 0.24 | 0.28 | 0.38 | 0.05 | 0.09 | 0.15 |
| ENSRNOG00000017878 | Aldh1a7 | 1 | 2.2E+08 | 2.2E+08 | - | 0.16 | 0.08 | 0.27 | 0.09 | 0.08 | 0.06 | 0.83 | 0.82 | 1.29 | 0.41 | 0.25 | 0.35 |
| ENSRNOG00000043093 | Ap1m2 | 8 | 2.0E+07 | 2.0E+07 | - | 0.00 | 0.00 | 0.00 | 0.26 | 0.09 | 0.04 | 0.21 | 0.20 | 0.03 | 0.00 | 0.00 | 0.00 |
| ENSRNOG00000042711 | Ccdc188 | 11 | 8.3E+07 | 8.3E+07 | - | 0.00 | 0.00 | 0.00 | 0.15 | 0.13 | 0.04 | 0.48 | 0.31 | 0.49 | 0.00 | 0.00 | 0.00 |
| ENSRNOG00000027936 | Ccdc8 | 1 | 7.8E+07 | 7.8E+07 | + | 0.00 | 0.00 | 0.00 | 0.02 | 0.01 | 0.03 | 2.81 | 3.33 | 2.87 | 0.00 | 0.00 | 0.00 |
| ENSRNOG00000013928 | Dsp | 17 | 2.7E+07 | 2.7E+07 | - | 0.06 | 0.04 | 0.04 | 0.27 | 0.25 | 0.34 | 0.91 | 1.04 | 0.76 | 0.10 | 0.13 | 0.10 |
| ENSRNOG00000025300 | Fer1l6 | 7 | 9.0E+07 | 9.0E+07 | + | 0.06 | 0.09 | 0.06 | 0.03 | 0.04 | 0.05 | 0.27 | 0.35 | 0.40 | 0.00 | 0.00 | 0.00 |
| ENSRNOG00000009751 | Gpx7 | 5 | 1.2E+08 | 1.2E+08 | - | 0.00 | 0.00 | 0.00 | 0.73 | 1.52 | 1.07 | 0.65 | 0.54 | 0.62 | 0.00 | 0.00 | 0.09 |
| ENSRNOG00000014117 | Hmox1 | 19 | 1.3E+07 | 1.3E+07 | + | 1053.62 | 987.69 | 994.77 | 52.14 | 51.54 | 55.70 | 13133.62 | 13381.25 | 14328.23 | 3522.95 | 3587.66 | 3752.48 |
| ENSRNOG00000025273 | L3mbtl4 | 9 | 1.1E+08 | 1.1E+08 | + | 0.00 | 0.00 | 0.00 | 0.03 | 0.02 | 0.03 | 0.31 | 0.39 | 0.42 | 0.00 | 0.00 | 0.00 |
| ENSRNOG00000068619 | LOC108353138 | 17 | 8.6E+07 | 8.6E+07 | - | 0.00 | 0.00 | 0.00 | 0.03 | 0.01 | 0.01 | 0.05 | 0.08 | 0.05 | 0.00 | 0.00 | 0.00 |
| ENSRNOG00000054809 | Lrpl1 | 16 | 5.6E+07 | 5.6E+07 | - | 3.31 | 3.19 | 3.81 | 0.33 | 0.44 | 0.38 | 18.99 | 22.54 | 22.98 | 1.19 | 1.28 | 1.20 |
| ENSRNOG00000001959 | Mx1 | 11 | 3.7E+07 | 3.7E+07 | - | 0.08 | 0.07 | 0.03 | 0.38 | 0.35 | 0.25 | 0.33 | 0.30 | 0.17 | 0.09 | 0.16 | 0.16 |
| ENSRNOG00000051372 | Mycn | 6 | 3.6E+07 | 3.6E+07 | - | 1.01 | 1.03 | 0.31 | 4.17 | 3.71 | 3.99 | 13.97 | 15.73 | 14.38 | 1.67 | 1.60 | 2.01 |
| ENSRNOG00000036837 | Nfe2 | 7 | 1.3E+08 | 1.3E+08 | - | 0.00 | 0.00 | 0.00 | 0.03 | 0.04 | 0.08 | 0.16 | 0.37 | 0.37 | 0.00 | 0.00 | 0.00 |
| ENSRNOG00000003018 | Olfml2b | 13 | 8.3E+07 | 8.3E+07 | + | 0.02 | 0.00 | 0.03 | 0.17 | 0.25 | 0.15 | 0.44 | 0.53 | 0.36 | 0.03 | 0.00 | 0.00 |
| ENSRNOG00000014948 | Osgin1 | 19 | 4.7E+07 | 4.8E+07 | + | 22.01 | 18.57 | 18.79 | 8.03 | 8.12 | 8.35 | 232.58 | 223.24 | 228.46 | 20.82 | 22.63 | 22.00 |
| ENSRNOG00000006280 | Pcsk9 | 5 | 1.2E+08 | 1.2E+08 | - | 0.00 | 0.00 | 0.00 | 0.18 | 0.27 | 0.33 | 0.26 | 0.27 | 0.39 | 0.16 | 0.11 | 0.07 |
| ENSRNOG00000023440 | Phex | X | 3.8E+07 | 3.8E+07 | + | 0.31 | 0.34 | 0.29 | 0.09 | 0.10 | 0.05 | 0.99 | 1.79 | 1.52 | 0.26 | 0.26 | 0.13 |
| ENSRNOG00000055293 | Ptprb | 7 | 5.2E+07 | 5.2E+07 | + | 0.00 | 0.00 | 0.00 | 0.03 | 0.02 | 0.01 | 0.13 | 0.09 | 0.08 | 0.00 | 0.00 | 0.01 |
| ENSRNOG00000005370 | S1pr4 | 7 | 8.2E+06 | 8.2E+06 | + | 0.02 | 0.05 | 0.04 | 0.16 | 0.17 | 0.29 | 0.64 | 0.58 | 0.68 | 0.02 | 0.09 | 0.06 |
| ENSRNOG00000036673 | Sectm1b | 10 | 1.1E+08 | 1.1E+08 | - | 0.05 | 0.08 | 0.00 | 0.37 | 0.27 | 0.48 | 0.47 | 0.47 | 0.49 | 0.00 | 0.08 | 0.00 |
| ENSRNOG00000010210 | Slc7a11 | 2 | 1.3E+08 | 1.3E+08 | - | 5.62 | 4.98 | 3.83 | 2.56 | 1.98 | 1.96 | 59.32 | 69.89 | 73.12 | 14.22 | 13.61 | 13.53 |
| ENSRNOG00000003537 | Spta1 | 13 | 8.6E+07 | 8.6E+07 | + | 0.13 | 0.18 | 0.17 | 0.05 | 0.11 | 0.13 | 1.57 | 2.01 | 1.74 | 0.84 | 0.78 | 0.89 |
| ENSRNOG00000003147 | Sqstm1 | 10 | 3.5E+07 | 3.5E+07 | - | 590.70 | 495.94 | 540.20 | 368.99 | 357.20 | 362.34 | 2712.26 | 2728.76 | 3017.07 | 983.66 | 976.21 | 974.46 |
| ENSRNOG00000054548 | Srpk3 | X | 1.5E+08 | 1.5E+08 | + | 0.00 | 0.00 | 0.00 | 0.13 | 0.17 | 0.13 | 0.25 | 0.12 | 0.11 | 0.00 | 0.02 | 0.00 |
| ENSRNOG00000027480 | Tmem116 | 12 | 3.5E+07 | 3.5E+07 | - | 0.57 | 0.52 | 0.24 | 0.18 | 0.12 | 0.17 | 2.10 | 2.62 | 3.74 | 0.46 | 0.29 | 0.36 |
| ENSRNOG00000028083 | Tmem35b | 5 | 1.4E+08 | 1.4E+08 | + | 0.00 | 0.00 | 0.00 | 0.10 | 0.08 | 0.16 | 1.60 | 1.04 | 0.74 | 0.02 | 0.02 | 0.00 |
| ENSRNOG00000048169 | Tuba8 | 4 | 1.5E+08 | 1.5E+08 | + | 0.14 | 0.16 | 0.41 | 0.06 | 0.06 | 0.03 | 0.96 | 1.06 | 0.74 | 0.00 | 0.00 | 0.00 |
| ENSRNOG00000009088 | Txnrd1 | 7 | 2.1E+07 | 2.1E+07 | - | 172.55 | 152.42 | 139.64 | 105.99 | 115.91 | 106.38 | 696.41 | 719.13 | 732.30 | 259.34 | 253.92 | 254.43 |
| ENSRNOG00000003845 | Wnt3 | 10 | 8.9E+07 | 8.9E+07 | + | 0.00 | 0.00 | 0.00 | 0.07 | 0.07 | 0.06 | 0.35 | 0.68 | 0.14 | 0.00 | 0.00 | 0.03 |

**Table S2. Intersection genes of differential expression (continued from the previous page).**

| **Gene ID** | **Gene Symbol** | **Comparison 1: WT+Hemin *vs.* WT** | | | | **Comparison 2: TRF-OE+Hemin *vs.* WT+Hemin** | | | |
| --- | --- | --- | --- | --- | --- | --- | --- | --- | --- |
|  |  | **log_2_(FC)** | ***P* Value** | ***P* Adjusted** | **Regulation** | **log_2_(FC)** | ***P* Value** | ***P* Adjusted** | **Regulation** |
| ENSRNOG00000028043 | Cxcl3 | -1.38 | 4.8E-06 | 1.3E-05 | Down | 1.30 | 9.2E-07 | 2.2E-06 | Up |
| ENSRNOG00000019500 | Cyp1a1 | -2.91 | 2.1E-28 | 2.9E-27 | Down | 3.40 | 4.7E-98 | 1.2E-96 | Up |
| ENSRNOG00000033202 | Fbxo41 | -1.28 | 9.9E-05 | 0.0002 | Down | -2.06 | 1.0E-09 | 3.1E-09 | Down |
| ENSRNOG00000030449 | Gsta4 | -1.83 | 1.8E-23 | 2.0E-22 | Down | 1.17 | 1.9E-83 | 3.8E-82 | Up |
| ENSRNOG00000057443 | Nos2 | -5.80 | 3.8E-69 | 2.3E-67 | Down | 3.61 | 3.8E-45 | 3.8E-44 | Up |
| ENSRNOG00000001369 | Oas1a | -1.67 | 0.0006 | 0.0013 | Down | 2.22 | 2.9E-07 | 7.3E-07 | Up |
| ENSRNOG00000066264 | Pilrb2l2 | -2.02 | 3.8E-16 | 2.6E-15 | Down | 1.37 | 8.3E-20 | 4.0E-19 | Up |
| ENSRNOG00000005302 | Slc2a9 | -1.21 | 7.8E-05 | 0.0002 | Down | -1.67 | 0.0003 | 0.0007 | Down |
| ENSRNOG00000017878 | Aldh1a7 | 2.00 | 0.0006 | 0.0012 | Up | -1.61 | 2.7E-06 | 6.2E-06 | Down |
| ENSRNOG00000043093 | Ap1m2 | 5.54 | 0.0007 | 0.0015 | Up | -5.90 | 0.0002 | 0.0005 | Down |
| ENSRNOG00000042711 | Ccdc188 | 7.45 | 6.1E-09 | 2.3E-08 | Up | -7.81 | 3.2E-10 | 9.9E-10 | Down |
| ENSRNOG00000027936 | Ccdc8 | 10.94 | 1.2E-19 | 1.0E-18 | Up | -11.31 | 1.8E-21 | 9.2E-21 | Down |
| ENSRNOG00000013928 | Dsp | 3.80 | 5.2E-35 | 9.8E-34 | Up | -3.10 | 1.1E-38 | 9.4E-38 | Down |
| ENSRNOG00000025300 | Fer1l6 | 1.80 | 1.7E-06 | 4.9E-06 | Up | -8.87 | 2.6E-13 | 9.5E-13 | Down |
| ENSRNOG00000009751 | Gpx7 | 7.42 | 2.0E-09 | 7.8E-09 | Up | -4.15 | 0.0044 | 0.0075 | Down |
| ENSRNOG00000014117 | Hmox1 | 3.28 | 1.3E-266 | 1.3E-263 | Up | -1.99 | 1.3E-278 | 1.6E-276 | Down |
| ENSRNOG00000025273 | L3mbtl4 | 7.88 | 2.1E-10 | 9.0E-10 | Up | -8.25 | 8.9E-12 | 3.0E-11 | Down |
| ENSRNOG00000068619 | LOC108353138 | 5.14 | 0.0002 | 0.0003 | Up | -5.50 | 2.2E-05 | 4.7E-05 | Down |
| ENSRNOG00000054809 | Lrpl1 | 2.16 | 2.1E-25 | 2.5E-24 | Up | -4.22 | 1.1E-297 | 1.6E-295 | Down |
| ENSRNOG00000001959 | Mx1 | 1.60 | 0.0028 | 0.0054 | Up | -1.09 | 0.0115 | 0.0188 | Down |
| ENSRNOG00000051372 | Mycn | 3.77 | 1.0E-13 | 5.7E-13 | Up | -3.14 | 6.9E-136 | 2.6E-134 | Down |
| ENSRNOG00000036837 | Nfe2 | 6.65 | 9.2E-07 | 2.7E-06 | Up | -7.02 | 7.3E-08 | 1.9E-07 | Down |
| ENSRNOG00000003018 | Olfml2b | 4.10 | 2.9E-08 | 9.8E-08 | Up | -5.44 | 1.6E-10 | 4.9E-10 | Down |
| ENSRNOG00000014948 | Osgin1 | 3.05 | 2.2E-233 | 2.1E-230 | Up | -3.45 | 0.0000 | 0.0000 | Down |
| ENSRNOG00000006280 | Pcsk9 | 7.50 | 3.5E-09 | 1.3E-08 | Up | -1.47 | 0.0003 | 0.0006 | Down |
| ENSRNOG00000023440 | Phex | 1.73 | 2.8E-07 | 8.7E-07 | Up | -2.77 | 5.8E-11 | 1.9E-10 | Down |
| ENSRNOG00000055293 | Ptprb | 7.77 | 1.4E-09 | 5.6E-09 | Up | -5.70 | 2.0E-09 | 5.8E-09 | Down |
| ENSRNOG00000005370 | S1pr4 | 3.53 | 1.7E-08 | 6.0E-08 | Up | -3.28 | 4.4E-11 | 1.4E-10 | Down |
| ENSRNOG00000036673 | Sectm1b | 2.75 | 5.1E-05 | 0.0001 | Up | -4.04 | 1.7E-06 | 4.1E-06 | Down |
| ENSRNOG00000010210 | Slc7a11 | 3.35 | 1.9E-77 | 1.5E-75 | Up | -2.37 | 1.6E-126 | 5.4E-125 | Down |
| ENSRNOG00000003537 | Spta1 | 2.99 | 1.2E-27 | 1.7E-26 | Up | -1.16 | 1.3E-17 | 5.6E-17 | Down |
| ENSRNOG00000003147 | Sqstm1 | 1.80 | 1.7E-47 | 5.3E-46 | Up | -1.59 | 1.4E-155 | 6.5E-154 | Down |
| ENSRNOG00000054548 | Srpk3 | 5.96 | 2.2E-05 | 5.5E-05 | Up | -4.46 | 0.0002 | 0.0005 | Down |
| ENSRNOG00000027480 | Tmem116 | 2.13 | 8.6E-07 | 2.5E-06 | Up | -2.94 | 8.0E-17 | 3.4E-16 | Down |
| ENSRNOG00000028083 | Tmem35b | 7.65 | 1.1E-08 | 4.1E-08 | Up | -6.15 | 5.6E-08 | 1.5E-07 | Down |
| ENSRNOG00000048169 | Tuba8 | 1.45 | 0.0173 | 0.0297 | Up | -8.59 | 1.6E-12 | 5.4E-12 | Down |
| ENSRNOG00000009088 | Txnrd1 | 1.74 | 3.8E-206 | 2.1E-203 | Up | -1.57 | 0.0000 | 0.0000 | Down |
| ENSRNOG00000003845 | Wnt3 | 7.66 | 8.7E-07 | 2.6E-06 | Up | -5.19 | 0.0003 | 0.0006 | Down |

Chromosome: Chromosome ID where gene is located. Start/End: The full-length start or termination site of gene in the genome. Strand: The feature of the strand in the gene; “+” indicates the sense strand, and “-” indicates the antisense strand. TPM: Transcripts per million. log_2_(FC): The log scale of fold change (the absolute ratio of normalized intensities between groups). *P* Value: Significance index of statistical difference. *P* Adjusted: The corrected *P* Value. Regulation: “Down” means differentially downregulated, and “Up” means differentially upregulated.

**Table S3. GO annotation of intersection genes.**

| **GO ID** | **GO Term** | **Description** | **Gene ID** | **Gene Count** | ***P* Value** | **Enrichment Score** |
| --- | --- | --- | --- | --- | --- | --- |
| GO:0071248 | Biological Process | cellular response to metal ion | Cyp1a1/Gsta4/Hmox1/Nos2/Txnrd1 | 5 | 6.4E-05 | 4.19 |
| GO:0009636 | Biological Process | response to toxic substance | Cyp1a1/Gpx7/Gsta4/Slc7a11/Txnrd1 | 5 | 1.6E-04 | 3.81 |
| GO:0051775 | Biological Process | response to redox state | Nos2/Slc7a11 | 2 | 1.9E-04 | 3.72 |
| GO:0072593 | Biological Process | reactive oxygen species metabolic process | Cyp1a1/Mycn/Nos2/Txnrd1 | 4 | 7.5E-04 | 3.12 |
| GO:0009069 | Biological Process | serine family amino acid metabolic process | Slc7a11/Txnrd1 | 2 | 1.9E-03 | 2.73 |
| GO:0010039 | Biological Process | response to iron ion | Cyp1a1/Hmox1 | 2 | 3.9E-03 | 2.40 |
| GO:0042743 | Biological Process | hydrogen peroxide metabolic process | Cyp1a1/Txnrd1 | 2 | 4.1E-03 | 2.39 |
| GO:0009064 | Biological Process | glutamine family amino acid metabolic process | Nos2/Slc7a11 | 2 | 6.9E-03 | 2.16 |
| GO:0006749 | Biological Process | glutathione metabolic process | Gsta4/Slc7a11 | 2 | 7.3E-03 | 2.14 |
| GO:0043122 | Biological Process | regulation of I-kappaB kinase/NF-kappaB signaling | Hmox1/Sectm1b/Sqstm1 | 3 | 8.3E-03 | 2.08 |
| GO:0030863 | Cellular Component | cortical cytoskeleton | Nos2/Spta1 | 2 | 1.8E-02 | 1.74 |
| GO:0005791 | Cellular Component | rough endoplasmic reticulum | Pcsk9/Slc7a11 | 2 | 1.9E-02 | 1.71 |
| GO:0032437 | Cellular Component | cuticular plate | Spta1 | 1 | 2.2E-02 | 1.66 |
| GO:0044754 | Cellular Component | autolysosome | Sqstm1 | 1 | 2.2E-02 | 1.66 |
| GO:0005767 | Cellular Component | secondary lysosome | Sqstm1 | 1 | 2.9E-02 | 1.54 |
| GO:0001533 | Cellular Component | cornified envelope | Dsp | 1 | 3.7E-02 | 1.43 |
| GO:0097449 | Cellular Component | astrocyte projection | Slc7a11 | 1 | 3.7E-02 | 1.43 |
| GO:0030057 | Cellular Component | desmosome | Dsp | 1 | 4.0E-02 | 1.40 |
| GO:0030135 | Cellular Component | coated vesicle | Ap1m2/Pcsk9 | 2 | 4.7E-02 | 1.32 |
| GO:0000407 | Cellular Component | phagophore assembly site | Sqstm1 | 1 | 5.3E-02 | 1.27 |
| GO:0030545 | Molecular Function | signaling receptor regulator activity | Cxcl3/Osgin1/Pcsk9/Sectm1b/Wnt3 | 5 | 1.8E-03 | 2.75 |
| GO:0004497 | Molecular Function | monooxygenase activity | Cyp1a1/Hmox1/Nos2 | 3 | 2.1E-03 | 2.68 |
| GO:0020037 | Molecular Function | heme binding | Cyp1a1/Hmox1/Nos2 | 3 | 3.0E-03 | 2.53 |
| GO:0051879 | Molecular Function | Hsp90 protein binding | Cyp1a1/Nos2 | 2 | 3.7E-03 | 2.43 |
| GO:0005125 | Molecular Function | cytokine activity | Cxcl3/Sectm1b/Wnt3 | 3 | 4.8E-03 | 2.32 |
| GO:0005080 | Molecular Function | protein kinase C binding | Dsp/Sqstm1 | 2 | 5.3E-03 | 2.28 |
| GO:0016705 | Molecular Function | oxidoreductase activity, acting on paired donors, with incorporation or reduction of molecular oxygen | Cyp1a1/Hmox1/Nos2 | 3 | 6.6E-03 | 2.18 |
| GO:0048018 | Molecular Function | receptor ligand activity | Cxcl3/Osgin1/Sectm1b/Wnt3 | 4 | 8.5E-03 | 2.07 |
| GO:0030546 | Molecular Function | signaling receptor activator activity | Cxcl3/Osgin1/Sectm1b/Wnt3 | 4 | 9.1E-03 | 2.04 |
| GO:0016209 | Molecular Function | antioxidant activity | Gpx7/Txnrd1 | 2 | 9.9E-03 | 2.00 |

The top 10 most significant enrichment terms in biological process, cellular component, and molecular function, respectively, of intersection genes in GO analysis. The Enrichment Score (-log10 scale of *P* Value) expressed the regulation extent of the predicted functions by the genes.

**Table S4. KEGG annotation of intersection genes.**

| **KEGG ID** | **Description** | **Gene ID** | **Gene Count** | ***P* Value** | **Enrichment Score** |
| --- | --- | --- | --- | --- | --- |
| rno04216 | Ferroptosis | Hmox1/Slc7a11 | 2 | 0.0031 | 2.51 |
| rno00480 | Glutathione metabolism | Gpx7/Gsta4 | 2 | 0.0073 | 2.14 |
| rno04066 | HIF-1 signaling pathway | Hmox1/Nos2 | 2 | 0.0315 | 1.50 |
| rno00220 | Arginine biosynthesis | Nos2 | 1 | 0.0358 | 1.45 |
| rno04621 | NOD-like receptor signaling pathway | Cxcl3/Oas1a | 2 | 0.0405 | 1.39 |
| rno04979 | Cholesterol metabolism | Pcsk9 | 1 | 0.0864 | 1.06 |
| rno04540 | Gap junction | Tuba8 | 1 | 0.1405 | 0.85 |
| rno04146 | Peroxisome | Nos2 | 1 | 0.1435 | 0.84 |
| rno04657 | IL-17 signaling pathway | Cxcl3 | 1 | 0.1465 | 0.83 |
| rno04064 | NF-kappa B signaling pathway | Cxcl3 | 1 | 0.1598 | 0.80 |
| rno04668 | TNF signaling pathway | Cxcl3 | 1 | 0.1873 | 0.73 |
| rno04926 | Relaxin signaling pathway | Nos2 | 1 | 0.2015 | 0.70 |
| rno04071 | Sphingolipid signaling pathway | S1pr4 | 1 | 0.2043 | 0.69 |
| rno04068 | FoxO signaling pathway | S1pr4 | 1 | 0.2112 | 0.68 |
| rno04390 | Hippo signaling pathway | Wnt3 | 1 | 0.2425 | 0.62 |
| rno04150 | mTOR signaling pathway | Wnt3 | 1 | 0.2451 | 0.61 |
| rno04310 | Wnt signaling pathway | Wnt3 | 1 | 0.2609 | 0.58 |
| rno04217 | Necroptosis | Sqstm1 | 1 | 0.2713 | 0.57 |
| rno04062 | Chemokine signaling pathway | Cxcl3 | 1 | 0.2725 | 0.56 |
| rno04020 | Calcium signaling pathway | Nos2 | 1 | 0.3595 | 0.44 |

All relevant enrichment terms of intersection genes in KEGG analysis. The Enrichment Score (-log10 scale of *P* Value) expressed the regulation extent of the predicted functions by the genes.
