# Supplementary material for: Transferrin restrains rather than drives neuronal ferroptosis after intracerebral haemorrhage
Source: Front Pharmacol. 2026 Oct 28;17:1945504. doi: 10.3389/fphar.2026.1945504 (PMC13752033; doi:10.3389/fphar.2026.1945504)
Supplement: Supplementary file 1 [file DataSheet1.zip › Supplementary Material/Supplementary Material 2.pdf]

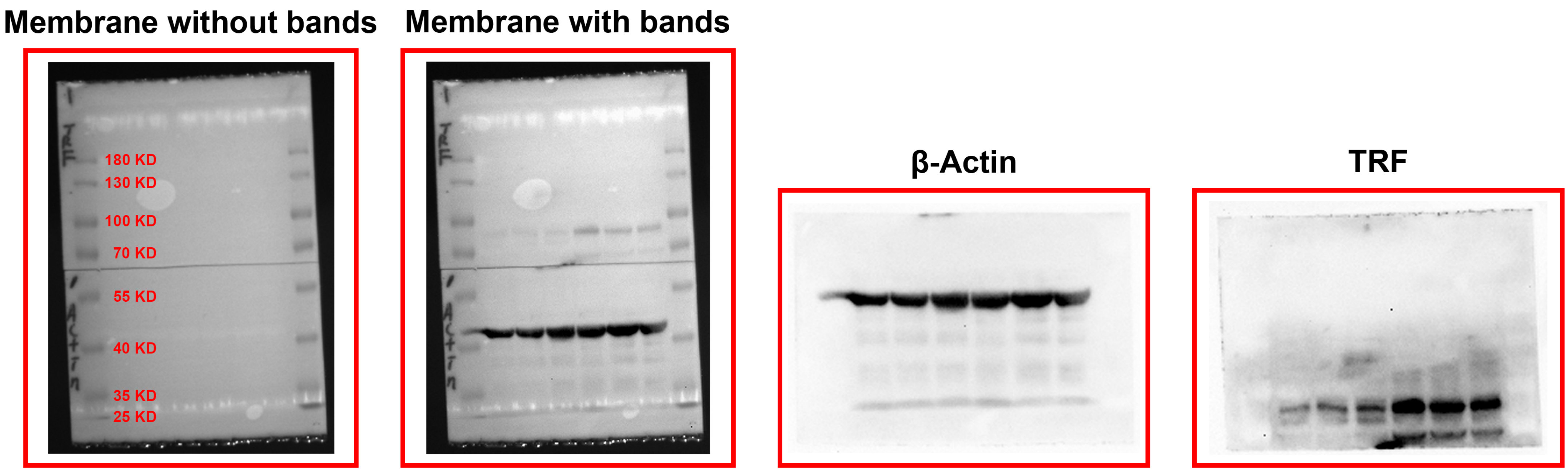
Membrane without bands
Membrane with bands
β-Actin
TRF
180 KD
130 KD
100 KD
70 KD
55 KD
40 KD
35 KD
25 KD
